# Untargeted LC-MS Metabolomics Differentiates Between Virulent and Avirulent Clinical Strains of *Pseudomonas aeruginosa*

**DOI:** 10.3390/biom10071041

**Published:** 2020-07-13

**Authors:** Tobias Depke, Janne Gesine Thöming, Adrian Kordes, Susanne Häussler, Mark Brönstrup

**Affiliations:** 1 Department of Chemical Biology, Helmholtz Centre for Infection Research, 38124 Braunschweig, Germany; tobias.depke@helmholtz-hzi.de; 2 Institute of Molecular Bacteriology, Twincore, Centre for Clinical and Experimental Infection Research, 30625 Hannover, Germany; janne.gesine.thoeming@regionh.dk (J.G.T.); adrian.kordes@twincore.de (A.K.); susanne.haeussler@helmholtz-hzi.de (S.H.); 3 Department of Molecular Bacteriology, Helmholtz Centre for Infection Research, 38124 Braunschweig, Germany; 4 German Centre for Infection Research (DZIF), Partner Site Hannover-Braunschweig, 38124 Braunschweig, Germany

**Keywords:** *Pseudomonas aeruginosa*, virulence, untargeted metabolomics, LC-MS, random forest classification, biomarker, phenotyping

## Abstract

*Pseudomonas aeruginosa* is a facultative pathogen that can cause, inter alia, acute or chronic pneumonia in predisposed individuals. The gram-negative bacterium displays considerable genomic and phenotypic diversity that is also shaped by small molecule secondary metabolites. The discrimination of virulence phenotypes is highly relevant to the diagnosis and prognosis of *P. aeruginosa* infections. In order to discover small molecule metabolites that distinguish different virulence phenotypes of *P. aeruginosa*, 35 clinical strains were cultivated under standard conditions, characterized in terms of virulence and biofilm phenotype, and their metabolomes were investigated by untargeted liquid chromatography—mass spectrometry. The data was both mined for individual candidate markers as well as used to construct statistical models to infer the virulence phenotype from metabolomics data. We found that clinical strains that differed in their virulence and biofilm phenotype also had pronounced divergence in their metabolomes, as underlined by 332 features that were significantly differentially abundant with fold changes greater than 1.5 in both directions. Important virulence-associated secondary metabolites like rhamnolipids, alkyl quinolones or phenazines were found to be strongly upregulated in virulent strains. In contrast, we observed little change in primary metabolism. A hitherto novel cationic metabolite with a sum formula of C_12_H_15_N_2_ could be identified as a candidate biomarker. A random forest model was able to classify strains according to their virulence and biofilm phenotype with an area under the Receiver Operation Characteristics curve of 0.84. These findings demonstrate that untargeted metabolomics is a valuable tool to characterize *P. aeruginosa* virulence, and to explore interrelations between clinically important phenotypic traits and the bacterial metabolome.

## 1. Introduction

The γ-proteobacterium *Pseudomonas aeruginosa* thrives in various aquatic and terrestrial habitats [1], as well as in multiple niches in the human host such as the lungs, eyes and ears [2,3,4]. Its affinity and ability to form biofilms on surfaces enables this bacterium to colonize burn wounds and the surfaces of invasive medical devices such as catheters and implants [5]. This wide niche variability, along with its ability to produce various virulence factors and evade the immune system through numerous mechanisms including biofilm formation renders it a clinically relevant, yet difficult-to-treat opportunistic pathogen [6]. *P. aeruginosa* belongs to the group of most dominant bacteria involved in hospital-acquired infections, comprising an estimated 10% of nosocomial infections in the EU [7]. In particular, *P. aeruginosa* is a major threat to artificially ventilated patients [8] and those with cystic fibrosis (CF), of which roughly 60% are chronically infected by *P. aeruginosa* [9].

*P. aeruginosa* displays high metabolic versatility, enabling it to infect and persist in different human tissues and organs [2]. As an example, it has been found that *P. aeruginosa* adapts its iron uptake strategies depending on the type of infection [10]. Nutrient availability in general differs between the various infection sites, and besides metabolism in the narrower sense, strategies of pathogenicity and persistence also need to be tailored to the specific infection situation. Many aspects of *P. aeruginosa*’s adaptability to different infection sites and types have been studied on the genomic level [11]. Genes coding for virulence factors are highly conserved among *P. aeruginosa* strains; however, there is little correlation between genomic features and the type of infection [12]. Despite a high conservation of virulence factors between clinical and environment samples [13], clinical *P. aeruginosa* strains have been demonstrated to express variable metabolomic, transcriptomic and phenotypic signatures despite almost identical genomes [14,15]. With respect to pathogenicity, clinical isolates can have vastly different phenotypes and elicit the full range of host responses [16,17].

Due to the diversity of genotypic and phenotypic traits, it is of considerable clinical interest to describe and differentiate the various metabolic and virulence properties of *P. aeruginosa* clinical isolates. At present, our understanding of genomics is insufficient to fully elucidate the metabolic and phenotypic variation of this bacterium at a clinically relevant level. Transcriptome data can be indicative of virulence phenotypes, yet not always sufficient, either, if used in isolation [18]. While numerous different phenotypic traits including, but not limited to, swimming motility have been proposed as virulence markers for *P. aeruginosa* clinical strains [19], several studies have suggested investigating metabolomes as functional signatures that might be closer to the actual phenotype [20,21,22]. In *P. aeruginosa*, many regulators and effectors of virulence are small-molecule secondary metabolites [23] that are generally amenable to liquid chromatography—mass spectrometry (LC-MS) [24,25,26]. Microbial metabolomics is becoming more prevalent in many areas of microbiology and infection research [27,28] and has demonstrated itself to be a successful methodology to, e.g., discriminate between different *Bacillus cereus* strains [29], to describe and differentiate drug susceptibility phenotypes in the parasite *Leishmania donovani* [30] as well as in the γ-proteobacterium *Acinetobacter baumannii* [31], to identify volatile metabolites in different *P. aeruginosa* strains [32] and to describe the metabolic adaptations of *P. aeruginosa* strains colonizing different niches in CF lungs [33].

Hence, we tested whether the virulence properties of *P. aeruginosa* clinical strains can be differentiated by untargeted LC-MS metabolomics. Metabolomics data for 35 clinical isolates recovered from diverse infection sites were acquired, stratified according to *in vivo* virulence phenotypes in the *Galleria mellonella* infection model [34] and biofilm phenotypes [15] and analyzed for discriminating markers. Data on the metabolite level and metabolic profiles were investigated, and a statistical model was generated to differentiate virulent and avirulent strains based solely on LC-MS data.

## 2. Materials and Methods

### 2.1. Bacterial Strains

Bacterial strains were selected from a biobank of *P. aeruginosa* clinical isolates curated at the Helmholtz Centre for Infection Research in Braunschweig, Germany, which is documented in the ‘Bactome’ database [35]. Strains were collected in clinical microbiology laboratories, private practice laboratories, or provided by strain collection curators across Germany, Spain, Hungary and Romania. Clinical isolates used in this study were previously characterized with regard to clinically relevant phenotypes [35,36,37], including *in vitro* biofilm phenotypes [15] and an *in vivo* virulence infection model using *Galleria mellonella* [34]. A list of strains and their phenotypic properties (biofilm and virulence) can be found in Table 1.

### 2.2. Transcriptomics

Transcriptional profiles of all clinical isolates used in this study were produced for a previous study [36]. Briefly, planktonic bacteria were cultivated to early stationary phase (OD_600_ = 2) in 10 ml LB under shaking conditions (37 °C, 180 rpm). We pooled three independent cultures to obtain one transcriptional profile per strain. cDNA libraries were generated using the ScriptSeq™ v2 RNA-seq Library Preparation Kit (Illumina), and samples were sequenced in single end mode on an Illumina HiSeq 2500 device (1 × 50 bp reads). The reads were mapped to the UCBPP-PA14 reference genome (NC_008463.1, available for download from the Pseudomonas Genome database: http://v2.pseudomonas.com) using the stampy pipeline [38]. RNA-Seq data of clinical isolates was uploaded at NCBI’s Gene Expression Omnibus (GSE123544). Differential gene expression analysis was performed using the R package DESeq2 (v.1.18.1) [39] with default settings to calculate the normalized reads per gene (nrpg). For the identification of differentially expressed genes between virulent and avirulent strains, a threshold of log_2_(fold change) ≥ 1 and ≤ −1 respectively with p_adj_ < 0.05 was applied. Only genes assigned to the core genes (according to Mathee et al. [40]) were considered for the analysis to account for differences in strain backgrounds (PA14 vs. PAO1). DESeq2 was used to generate a principal component analysis (PCA) plot from the transcriptional profiles.

### 2.3. Untargeted Metabolomics

All chemicals and analytical standards used in the metabolomics experiments in this study correspond to those previously described [24]. Selected strains were cultivated and measured in two distinct and independent batches, a discovery and a validation batch (cf. Table 1). For data analysis, the validation batch was divided into two sub-batches: the actual validation set containing strains with phenotypes that were also present in the discovery batch, and an additional set of isolates with virulent phenotypes containing the cluster C biofilm phenotype that was not present in the discovery batch. With this setup, the actual validation set could gauge the performance and validity of the applied statistical classification models (see below), because it contained strains that should be classified into the same categories as the strains in the discovery data set. As the phenotypes in the discovery data set were defined by virulence as well as biofilm morphology, an additional set of strains with a different combination of these two properties was needed to assess whether the model was able to differentiate solely the virulence phenotype irrespective of biofilm morphology.

Overnight precultures grown in 3 mL LB medium in glass tubes were inoculated from plate cultures for each strain and incubated for approximately 16 h at 37 °C and 140 rpm in a shaking incubator. Three independent biological replicates were inoculated with a starting OD_600_ of 0.05 from each preculture. Cultures were subsequently grown to an OD_600_ of approximately 2. Measured OD_600_ values for each sample were later used for normalization and can be found in Tables S1–S3. 2 mL of each sample was collected and immediately centrifuged at 9000× *g*, at 4 °C for 5 min. Cell pellets were snap-frozen in liquid nitrogen and subsequently stored at –20 °C until all of the batch samples were processed to this stage.

Metabolite extraction was performed as previously [24]. In brief, cell pellets were extracted in 500 μL methanol containing 0.1 mg/L trimethoprim, 0.1 mg/L nortriptylin and 0.3 mg/L glipizide as internal standards through the use of vigorous shaking and sonication. Extracts were separated from solid matter by centrifugation. 400 μL of each extract was concentrated to dryness and resuspended in 40 μL 50% (*v*/*v*) acetonitrile with 0.1% formic acid containing 1 mg/L caffeine and 8 mg/L naproxen as internal standards.

A 1 μL aliquot of each sample was analyzed by reversed phase ultra-high performance liquid chromatography coupled to quadrupole time-of-flight mass spectrometry as previously described [24,41]. Tandem mass spectra were recorded from pooled quality control samples and used for metabolite identification by comparison to authentic standards and/or metabolite databases as described in a previous study [24] (cf. Table S4). LC-MS data were exported to mzXML using Bruker Compass Xport, and preprocessed with XCMSonline [42] with the parameters listed in Table S5. Our discovery, validation and additional data sets were all processed separately.

After preprocessing, the untargeted metabolomics data underwent further processing using R/RStudio with ‘tidyverse’ packages as statistical software [43,44,45]. First, features eluting at retention times ≤ 0.8 min and ≥ 20 min, those displaying a relative standard deviation of ≤ 20% over all samples, and those with an intensity of ≤ 10,000 counts were removed. Subsequently, the data was consecutively normalized through the use of internal standards; first with those added upon reconstitution (caffeine and naproxen), and then with those added during extraction (trimethoprim and nortriptyline). The data for each sample was further normalized through the use of the respective OD_600_ at harvest as a proxy for cell number Tables S1–S3). Annotations were added and isotope peaks were identified using ‘CAMERA’ [46] (as part of the XCMSonline workflow) and removed from the data sets. The resulting feature tables for the discovery and the validation data sets were used for data analysis and model building.

Feature credentialing by means of stable isotope enriched growth medium [47] was performed in a previous study [48] and used to verify the biological origin of a candidate biomarker.

The Mass Spectrometry Search Tool (MASST) on the Global Natural Products Social Molecular Networking (GNPS) repository was used to match unidentified spectra of particular interest to previously reported MS^2^ data [49]. The standard parameters of the search were used: MS^2^ fragment ions were excluded if their *m/z* difference to the precursor ion was less than 17 Da and spectra were filtered using an approach called window filtering that keeps the 6 most abundant fragment ions within a ± 50 Da window throughout the spectrum. The *m/z* tolerance of the search was 2 Da for the precursor ion and 0.5 Da for MS^2^ fragment ions. To be considered a match, the queried spectrum and library spectra had to display a cosine similarity score of ≥ 0.7 and ≥ 6 matched peaks.

The raw data was uploaded to MetaboLights [50] and can be accessed via the study identifier MTBLS1749.

### 2.4. Data Analysis and Model Building

PCA was used with centering and rotating of the variables, and PCA scores were plotted for data exploration. Directional fold changes were calculated for all features with positive values signifying higher abundances in the virulent group and negative values signifying higher abundances in the avirulent group. Statistical significance of between-group differences was assessed by performing a Benjamini–Hochberg corrected two-sided Welch’s t-test for each feature. The Mann–Whitney U-test (Wilcoxon rank-sum test) was used to test for statistical significance for individual comparisons when non-normality was suspected. Using the ‘vegan’ R package [51], permutational multivariate analysis of variance (PERMANOVA) of a Bray-Curtis distance matrix of the metabolite abundance data was employed to test the correlation of metabolite profile to the phenotypic group. The same package was used to calculate the Shannon index to gauge differences in metabolome diversity between the samples of the different datasets.

Predictive models were built using random forest classification and the ‘randomForest’ R package [52,53] with 1000 trees per forest, and 500 randomly sampled variables considered as candidates at each split. Feature importance was assessed by mean increase of the Gini coefficient and mean increase of variable importance (VIP).

Model validation was performed by matching features of the discovery and validation data set and subsequent prediction of the phenotypes for all samples of the validation data set in the form of probabilities. The features were matched by comparing *m/z* and retention time, using a tolerance of 5 ppm and 1 min, respectively. Only features present in the discovery data set that matched a feature in the validation data set were considered in model building and validation. The same procedure was applied for the additional data set. Model quality was assessed by calculation of the area under the receiver operating characteristics (ROC) curve (AUC), using the ‘ROCR’ R package [54], where 1 corresponds to a perfect model and 0.5 is equivalent to random prediction. As the additional cluster C data set contains only one group of isolates, it was not possible to construct a ROC curve. Instead, the frequency of correct predictions for the strains in 100 independent constructions and predictions by the random forest model was assessed. This was also done for the first validation set.

## 3. Results

**Scheme 1:**
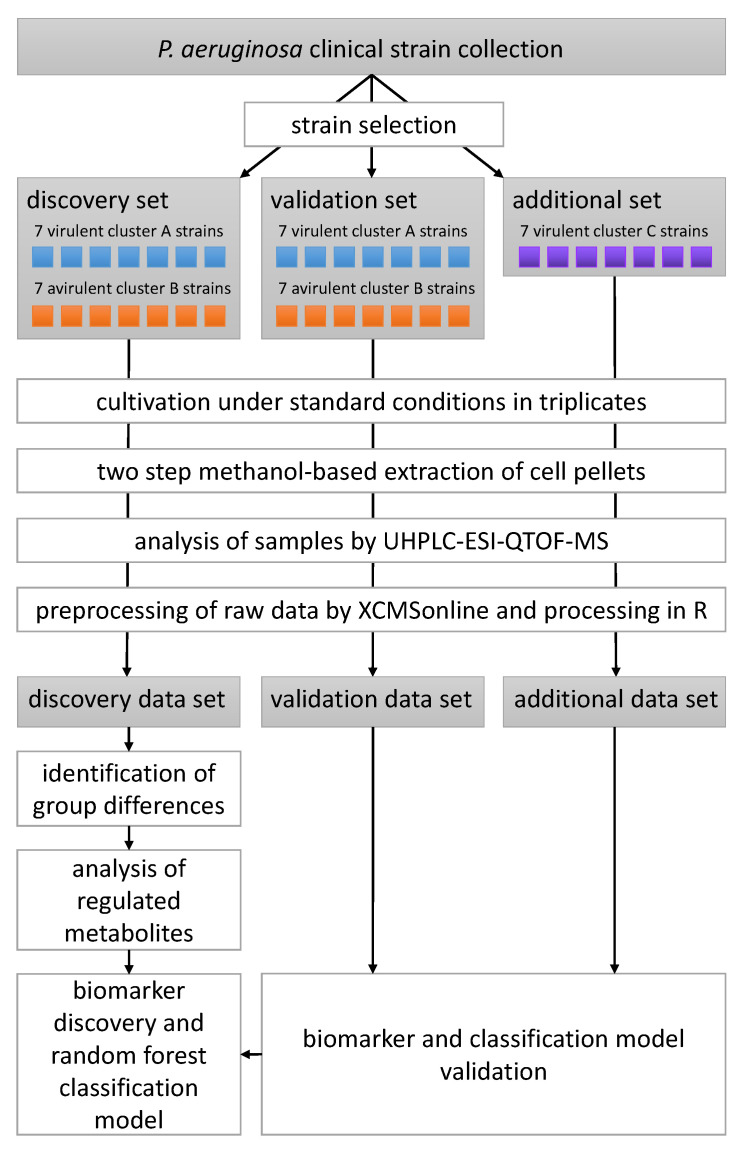
Schematic of the experimental and data analysis workflow of the metabolomics part of this study.

Untargeted LC-MS metabolomics data were recorded and analysed for 35 *P. aeruginosa* clinical strains differing in their virulence as determined by a *G. mellonella* survival model and their biofilm phenotype which was categorized into the three main clusters A, B and C [15]. A total of 14 strains, seven virulent strains with a cluster A biofilm phenotype and seven avirulent cluster B strains, constituted the discovery data set, that was analysed in depth for metabolomic differences between the phenotypes and used to build a random forest classification model. Another 14 strains equally split between the two phenotypic groups represented in the discovery group served as the validation data set. These strains were used to test the model constructed from the discovery data set. An additional seven virulent strains corresponding to a tertiary cluster C biofilm phenotype—i.e., a phenotype which was not present in the discovery data set—were used to investigate whether our classification model is capable of predicting virulence in a biofilm phenotype independent manner. All strains were cultivated in rich medium under standard planktonic conditions, extracted using a methanol-based protocol, separated on a reversed-phase C18 column and detected using time-of-flight mass spectrometry following electrospray ionization in positive mode (ESI-QTOF-MS). The study design is visualized in Scheme 1.

### 3.1. Virulent Cluster A and Avirulent Cluster B Strains Have Different Metabolic Profiles

**Figure 1:**
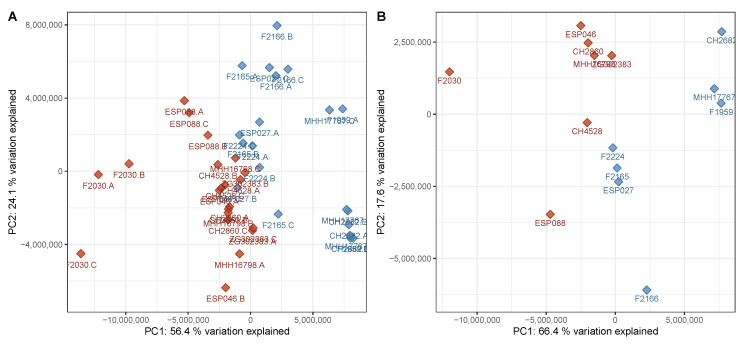
Principal component analysis (PCA) scores plot of the discovery data set. (**A**) All samples in the discovery data set were subjected to a principal components analysis with centering and rotation of the variables. The samples were plotted using principal components 1 and 2 as coordinates. A good separation of the groups is visible, predominantly in principal component 1, which explains 56% of the variation in the data set. (**B**) The data from the triplicate samples was averaged per strain and subjected to a PCA as described above. The separation is very similar to the data on the sample level. A clear separation can be achieved by considering both principal components 1 and 2. Red—virulent cluster A strains, blue—avirulent cluster B strains.

Overall variation in the untargeted metabolomics dataset (differences of signal abundances between and within groups after normalization and filtering with respect to all detected signals) was assessed using PCA. As an unsupervised method, PCA does not use class information, thereby preventing potential bias when judging separation between sample groups. Upon visual inspection, the PCA scores plot of the discovery data set, i.e., the data from the set of *P. aeruginosa* strains used to generate the classification model, showed a good but not complete separation between virulent cluster A and avirulent cluster B strains (Figure 1). Although the two phenotypes did not form compact clusters, there was little overlap between virulent and avirulent strains. Clear separation in an unsupervised analysis suggested that there is potential for a supervised method to model the data in a superior manner. A PERMANOVA analysis further supported the notion that the metabolome differences were associated with the virulence and biofilm phenotype (*F* = 10.7, *p* = 0.001). Moreover, the strains did not cluster according to other parameters such as time to reach the specified OD_600_, or the hospital they were originally isolated in (Figure S1). These findings suggest that, among our available metadata on the utilized *P. aeruginosa* isolates, virulence and biofilm phenotypes were the main drivers of variation between metabolomes.

The discovery data set contained 2359 features, whereof 135 were structurally annotated that corresponded to 96 unique metabolites. 332 features were significantly differentially abundant with fold changes greater than 1.5 in both directions as well as with a Benjamini–Hochberg corrected *p*-value of less than 0.05. 299 of these features were more abundant in the virulent group and 56 of the 332 (17%) were structurally annotated (cf. Figure S2), corresponding to 40 unique metabolites.

**Figure 2:**
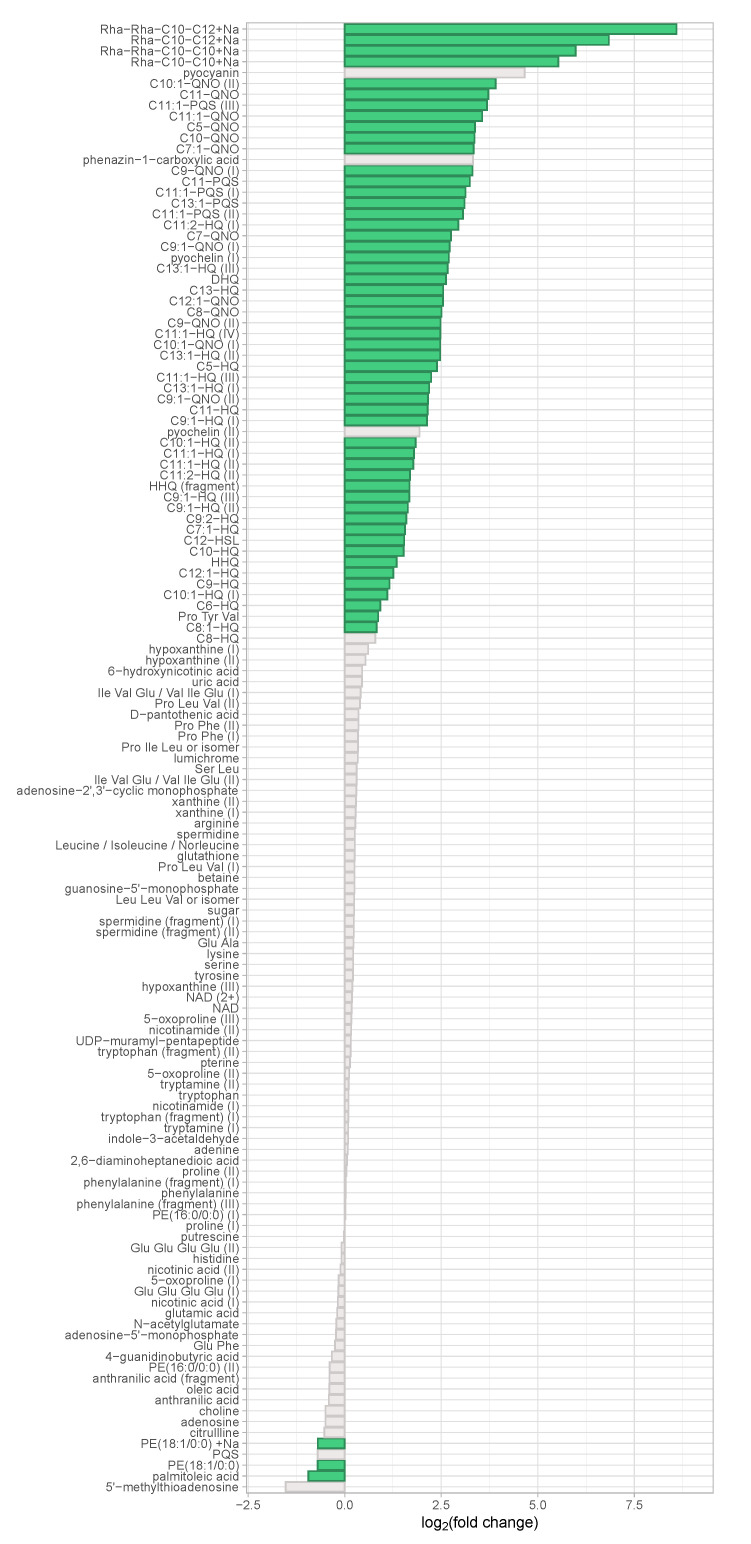
Regulation of identified metabolites in the discovery data set. The binary logarithm of their (non-directional) fold change was plotted on the x-axis for all identified metabolites. Features exhibiting a fold change ≥ 1.5 and a corrected *p*-value ≤ 0.05 were coloured in green.

**Figure 3:**
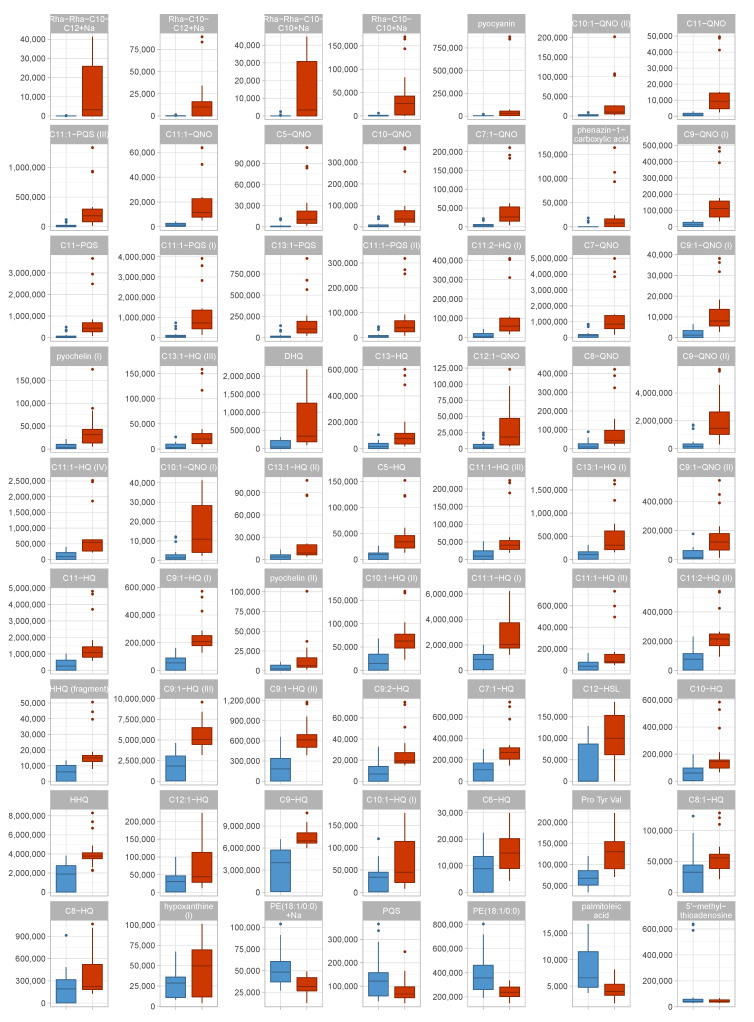
Box plots of identified metabolites in the discovery data set. Data distribution for all identified metabolites with an absolute fold change of ≥ 1.5 are shown as box plots. For each metabolite, one boxplot shows the abundances in each group. Red—virulent cluster A strains, blue—avirulent cluster B strains.

Among the identified metabolites with differential abundance, secondary metabolites are found along with lipids. Secondary metabolites were discovered at higher levels, while lipids were less abundant, in the virulent strains (Figure 2 and Figure 3). Virulent and avirulent *P. aeruginosa* strains did not differ in their relative abundances of primary metabolites. A PCA scores plot considering only identified metabolites provides good separation between the groups that were tested (Figure S3). Additionally, the PCA loadings plot of the complete discovery set, to which degree features contribute to the principal components, provides evidence that most features with high loadings have been annotated (Figure S4). These two observations demonstrate that most relevant metabolites, or at least members of the most relevant metabolite families, have been annotated. Interestingly, a PCA plot based on gene expression profiles did not show any clustering of the isolates according to their affiliation to a particular virulence phenotype (Figure S5).

### 3.2. Metabolic Differences Between Virulent Cluster A and Avirulent Cluster B Strains Manifest in Differential Abundance of Virulence-Associated Secondary Metabolites

Phenazines, prominent pseudomonal secondary metabolites with well-studied roles in pathogenesis and host cell damage, act as signaling molecules for the transcription factor SoxR which promotes the production of efflux pumps and the depletion of glutathione leading to redox instability of the host [55,56]. In this study, two phenazines were identified: pyocyanin and its congener phenazine-1-carboxylic acid. When comparing our virulent strains to our avirulent strains, both metabolites exhibited large differences in abundance with fold changes of +25 and +10, respectively. However, the difference between our virulent and avirulent strains was not statistically significant if tested using a Benjamini–Hochberg corrected Welch’s t-test due to the large variation within the virulent group containing two high producing strains, while all of the other strains produced much more modest phenazine levels (Figure S6). A non-parametric significance assessment using the Mann–Whitney U-test yielded *p*-values of $2.6\times 10^{-7}$ and $9.5\times 10^{-5}$ for pyocyanin and phenazine-1-carboxylic acid, respectively, thus suggesting significant differences in the abundance of phenazines in the two groups. Phenazine biosynthesis was also highly upregulated at the transcriptional level in virulent *P. aeruginosa* strains in comparison to the avirulent clinical isolates tested (Table S6), supporting the notion that phenazine production was associated with a virulent phenotype.

Alkyl quinolones (AQs), important quorum sensing signaling molecules unique to *P. aeruginosa* and closely related species, are involved in various virulence-associated processes [23]. Transcriptional profiles tend to provide evidence of the elevated expression of genes involved in the AQ biosynthesis in virulent isolates, however, the expression levels are not statistically significant (threshold log_2_(fold change) ≥ 1 and ≤ –1 with p_adj_ < 0.05) between the two groups (Table S7). Strikingly, the abundance of AQs in the metabolome of virulent *P. aeruginosa* strains is much greater than in the metabolome of the avirulent strains (Figure 2). For the highly abundant and important AQs HHQ (C7-HQ) and HQNO (C7-QNO), directional fold changes of +2.5 and +6.7 and corrected *p*-values of 0.0002 and 0.007, respectively, were observed. The most differentially abundant AQ is C10:1-QNO (directional fold change +15, corrected *p*-value 0.04), a metabolite with very low abundance (roughly 40× and 100× lower levels than C7-QNO in virulent cluster A and avirulent cluster B strains, respectively). The most significant difference was recorded for C9:1-HQ which was 3.2× more abundant in virulent cluster A strains with a corrected *p*-value of $3.1\times 10^{-6}$. The various AQ congeners detected in this study consistently showed significantly higher levels in the virulent group, had high loadings in the PCA and were good predictors in the random forest classification models, thereby emphasizing their importance in the regulation of virulence.

The largest fold changes between our virulent and avirulent strains were found in the rhamnolipids, another class of virulence-associated secondary metabolites (Figure S7). These surface-active glycolipids play multiple roles in the establishment and maintenance of infection, including the transition between biofilm and planktonic lifestyle [57] and the impairment of the host airway epithelium [58]. Rhamnolipids enable the poorly water-soluble *Pseudomonas* quinolone signal (PQS) to diffuse in aqueous environments, as they enhance the solubility of PQS through their amphiphilic properties, thereby potentiating PQS-driven effects on virulence [59]. We annotated four different rhamnolipid structures; namely Rha-Rha-C10-C12, Rha-Rha-C10-C10, Rha-C10-C12 and Rha-C10-C10 (Figure S8), and all of them were significantly more abundant in the virulent group (fold changes of +386, +63, +46, +114, respectively and corrected *p*-values of 0.001, 0.001, 0.01 and 0.02, respectively; always for Na adduct). In most avirulent strains, rhamnolipids were practically absent; likewise, some virulent strains barely produced any rhamnolipids, while others produced highly elevated levels of this secondary metabolite (Figure S7): For Rha-Rha-C10-C12, all avirulent cluster B strains and the virulent cluster A strains F2030, ESP088 and CH4528 showed a peak area in arbitrary units below 100, whereas the other virulent cluster A strains featured peak areas ranging from approximately 3000 to 40,000.

The pseudomonal siderophore pyochelin exists in *trans* and *cis* isoforms [60], both of which have been annotated. Interestingly, only one of the species was significantly regulated, and more prevalent in the virulent strains (directional fold change +6.5, corrected *p*-value 0.006). Pyochelin, an important player in iron acquisition and homeostasis, has been linked to virulence, although it is not necessarily directly harmful to the host [10]. Despite the significant differences in the amount of rhamnolipids and pyochelin produced by virulent strains over avirulent strains, the corresponding genes for both rhamnolipid and pyochelin biosynthesis were not differentially expressed between these two groups (Table S7).

Multiple primary and intermediate metabolites have been associated with pseudomonal virulence; however, no clear trends could be identified in the present study. For instance, tryptophan and phenylalanine, both of which share biosynthetic pathways with alkylquinolones and phenazines through the common precursor chorismate and are known inducers of PQS production [61], were not differentially abundant in the two groups (directional fold changes +1.1 and ±1.0, corrected *p*-values 0.5 and 0.9, respectively). Anthranilic acid, which is closely connected to the biosynthesis of phenazines, was also not significantly differentially abundant between our virulent and avirulent strains (directional fold change –1.3, corrected *p*-value 0.4).

**Figure 4:**
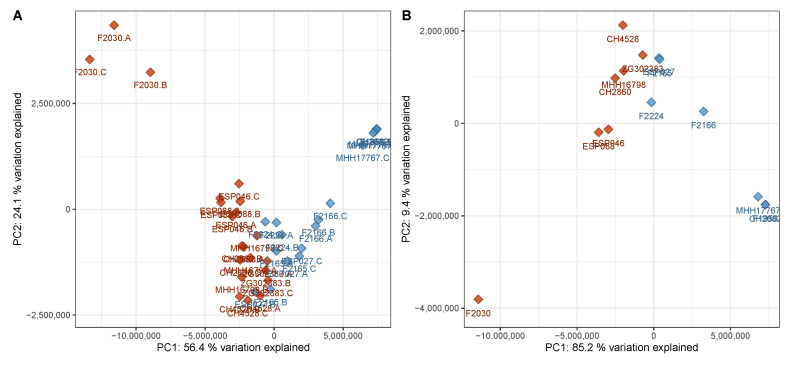
PCA scores plot of the discovery data set with only virulence-associated secondary metabolites included into the calculation. The plot has been created analogously to Figure 1 and shows a comparably good separation between the two phenotypes based on virulence-associated secondary metabolites only. (**A**) Data on sample level, (**B**) data on strain level (cf. Figure 1). Red—virulent cluster A strains, blue—avirulent cluster B strains.

When only identified metabolites that are known to be virulence-associated—AQs, DHQ, homoserine lactones, pyochelin, phenazines and rhamnolipids—were considered, a good separation between cluster A and cluster B was still visible in the PCA scores plot (Figure 4).

Figure 4 reveals that the metabolic profiles of one strain, F2030, differed from those of the other strains of the virulent cluster A group. This strain produced even higher AQ levels than the other virulent strains but displayed lower levels of other virulence-associated metabolites. Compared to the other samples of the same phenotype, F2030 sample harbored 2.1× more HHQ, 5.6× more C11-QNO and 3.6× more DHQ, but 2.2× less C12-HSL, 20× less Rha-C10-C10 and 27× less pyocyanin. The trends for the respective congeners were consistent.

### 3.3. An Unknown Metabolite Is a Potential Biomarker for Virulent Phenotypes

**Figure 5:**
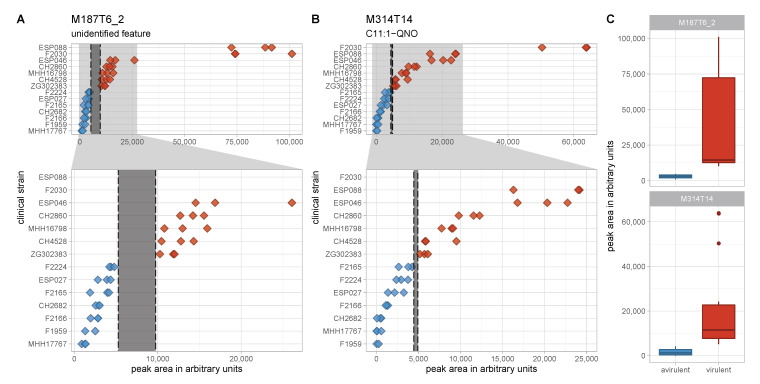
Levels of the two non-overlapping features in the discovery data set. (**A**,**B**) Peak area in arbitrary units is plotted on the x-axis for the two perfectly separating features M187T6_2 (**A**) and M314T14 (**B**). Full range of the x-axis is shown in the top panels and an enlarged region of the lower x-axis range is depicted in the lower panels. Non-overlapping spaces are marked by dashed lines and grey shading. The difference between the lowest level in the virulent cluster A group und the highest level in the avirulent cluster B group is a lot more pronounced for M187T6_2. (**C**) Peak area in arbitrary units is plotted on the y-axis for the two perfectly separating features M187T6_2 and M314T14 as box plots to visualize data distribution. Red—virulent cluster A strains, blue—avirulent cluster B strains.

In the search for classification biomarkers, the most interesting features are those that enable a clear group separation. In the discovery data set, there were only two features whose maximum levels in one group were lower than the minimum levels in the other group, thus allowing for an intensity threshold for the respective features to separate the groups completely (Figure 5). One is M314T14, putatively identified as C11:1-QNO, a relatively low abundant AQ. As all other AQ congeners show overlapping levels, M314T14 is most probably not a robust separator. The second feature, M187T6_2, however, has a larger non-overlapping intensity space between the two groups and thus appears to be a more promising separator. This feature exhibited a directional fold change of +11.7 and a Benjamini–Hochberg corrected *p*-value of 0.003. Statistical significance is also suggested by a *p*-value of $7.4\times 10^{-12}$ determined by the non-parametric Mann–Whitney U-test.

Despite considerable efforts, the identity of the feature could not be revealed by annotation strategies from the mixture, and efforts to purify the compound from raw extracts failed due to its very low abundance. Nonetheless, it could be demonstrated by means of feature credentialing [47] that the feature represents a metabolite produced by *P. aeruginosa* as it incorporated ^13^C from ^13^C_6_-glucose if supplied to the growth medium (Figure S9) [48]. Exact *m/z* and isotopic pattern analysis suggested the sum formula C_12_H_15_N_2_ for the positively charged ion. The MS^2^ spectrum of the feature was rather uninformative due to very weak fragmentation (Figure S10). However, the most dominant fragment peaks (relative intensity compared to base peak > 5%) displayed *m/z* ratios that supported the aforementioned sum formula, with an *m/z* of 145.076 corresponding to C_9_H_9_N_2_, an *m/z* of 144.068 to C_9_H_8_N_2_, and an *m/z* of 91.054 to C_7_H_7_—all possible fragments of C_12_H_15_N_2_. This formula is consistent with a reduced phenazine structure, namely hexahydrophenazine. A MASST search for similar MS^2^ with the same precursor in the GNPS data base [49] revealed that the feature has been detected in three other mass spectrometry studies that examined *P. aeruginosa* samples or bacterial samples from patients infected with CF (data sets MSV000080397, MSV000080337, MSV000080251, and MSV000079680, accessible at https://gnps.ucsd.edu/). Although none of studies found a meaningful annotation for the feature in question, its presence in other *P. aeruginosa* related data sets further supported the notion that it was an actual pseudomonal metabolite rather than an artifact.

The feature’s intensity levels did not correlate with those of pyocyanin or phenazine-1-carboxylic acid and correlate only weakly or insignificantly with rhamnolipids, C12-HSL or aromatic amino acids like phenylalanine, tryptophan or anthranilic acid. However, they did display a strong and significant correlation with AQs and pyochelin (Figure S11). The strongest (positive) correlation to an annotated feature was with DHQ (Pearson’s correlation coefficient of +0.93), suggesting a potential link to AQ biosynthesis. The strongest negative correlation to an annotated feature, in turn, was observed for adenosine, but appeared uninformative with a weak Pearson’s correlation coefficient of –0.47 despite its statistical significance.

M187T6_2 was tested as a potential marker for the differentiation of virulent and avirulent strains using a validation data set of another 14 clinical isolates—seven virulent and seven avirulent, displaying the same biofilm phenotype as those in the discovery data set—that was processed and analyzed analogously to the discovery data set. The feature corresponding to M187T6_2 in the validation set was significantly differentially abundant between the two virulence phenotype groups with a directional fold change of +3.6 and a *p*-value of 0.005 (Welch’s t-test) and 0.002 (Mann–Whitney U-test); however, it was unable to perfectly separate the groups (Figure S12). This is in concordance with higher metabolome diversity of the clinical strains (mean Shannon index of the samples in the discovery and validation data set: 5.93 and 6.25, respectively; *p* < 10^–17^), as illustrated by a more diffuse distribution of the virulent and avirulent strains in the PCA of the validation data set (Figure S13). A univariate model using the abundance of M187T6_2 as a separator for virulent and avirulent phenotypes yielded a fair area under the ROC curve of 0.75 (Figure S14). The feature in question did not display an intra-group correlation with the surrogate parameter used for *in vivo* virulence in this study—the survival of infected *Galleria mellonella* larvae after 48 h (Figure S15).

### 3.4. Virulent and Avirulent Strains with Distinct Biofilm Phenotypes Can Be Differentiated Based on Untargeted Metabolomics Data by Machine Learning

Since neither the single putative marker M187T6_2, nor any other metabolite could achieve a perfect group separation in the validation set, we tested whether a multi-metabolite classification model was able to reliably discriminate virulent and avirulent strains with their respective biofilm phenotypes. Random forest classification was selected from the plethora of machine learning classification models [62] as it did not require data to be on the same scale, and allowed for easy interpretation of the features’ contribution to the model. Random forest classification has also been shown to be a powerful method for phenotype discrimination based on clinical metabolomics data [63].

A random forest model was trained using the discovery data set, regarding only features that have been found and integrated both in the validation and discovery data set. The model was able to discriminate the groups in the discovery data set very clearly (Figure S16). Unsurprisingly, the M187T6_2 feature described above was the most important feature in the model. Among the 10 most important features were also C9-QNO and two isomers of C9:1-HQ, placing three AQ features in the top 10 of separating markers in the model. The remaining six of the most important features could not be identified. These include features with low *m/z* (M85T1_1 and M126T1_1), features in the *m/z* range of AQs and other secondary metabolites (M231T7_3, M246T3_1 and M228T12) and a slightly larger one (M464T9_3) (Figure S17). In a model based only on identified features, AQ congeners make up eight of the 10 most important features along with Rha-Rha-C10-C12 and pyocyanin.

**Figure 6:**
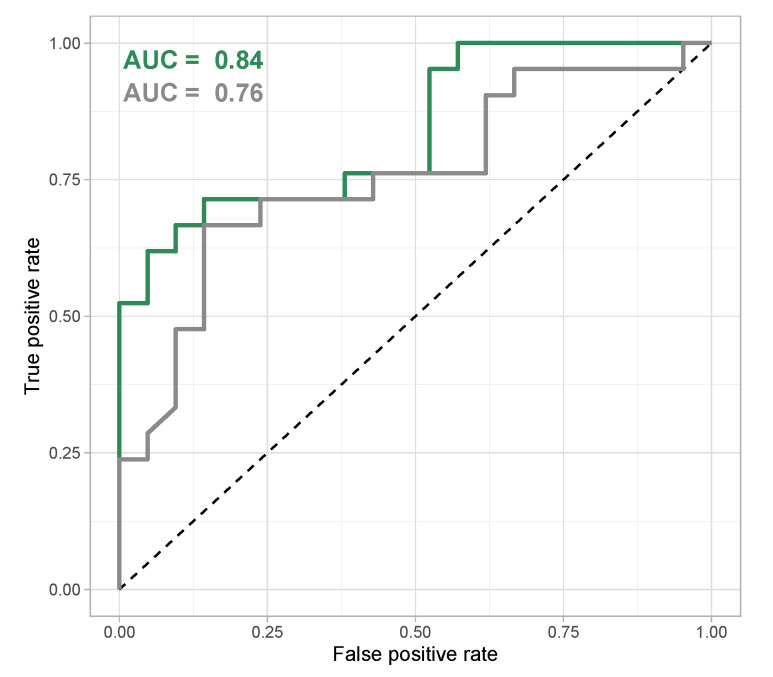
Receiver Operation Characteristics (ROC) curve for random forest classification model based on all features (green) and on identified features only (grey). The area under the Receiver Operation Characteristics curve is used as a quality metric for classification models and shows a good classification for a random forest classification model that uses all features in the data set, compared to a slightly weaker performance for a model that only considers identified features.

The classification model was applied to the validation data set to gauge its capacity to correctly predict virulence phenotypes from new metabolomics data obtained from *P. aeruginosa* clinical strains. The model showed a good prediction performance—especially regarding the larger heterogeneity of the validation data set—as signified by an area under the ROC curve of 0.84 (Figure 6). If only identified features were regarded, the area under the ROC curve of 0.76 was still fair, but including unknown features improved the prediction performance of the model. Remarkably, some strains were systematically misclassified (their metabolomes did not correspond to their virulence and biofilm phenotype in the way the classification model connected these two types of data) (Figure S18).

As the isolates of the discovery and validation data set differed in two phenotypes, virulence and biofilm phenotype, we analyzed a third group consisting of isolates that had a virulent phenotype in the *G. mellonella* model but a different (cluster C) biofilm morphology (Figure S19). Applying the random forest model to this group of isolates resulted in a true positive rate of only 47% (Figure S20) suggesting that despite a similar virulence phenotype, cluster A and cluster C isolates differed significantly in their metabolome.

## 4. Discussion

The present study provides evidence of systematic differences in the virulence-associated metabolome of several clinical *P. aeruginosa* strains isolated from several different types of infections in different hospitals across Europe. The metabolic profiles of virulent and avirulent strains with cluster A and cluster B biofilm phenotypes, respectively, in the discovery data set were sufficiently different to identify a separation between the two groups, even with an unsupervised method such as PCA, while other strain or cultivation-related properties were unable to separate these two groups.

Very few primary metabolites were significantly differentially abundant between the virulent cluster A and the avirulent cluster B groups, corroborating data from previous studies demonstrating that the primary metabolome of *P. aeruginosa* strongly depends on growth conditions, and weakly on the strain or genetic background [64]. Of the 43 identified distinct primary metabolites (cf. Table S8), only 11 had significantly different levels in the two groups in the discovery data set (with an adjusted *p*-value of ≤ 0.05), and only palmitoleic acid and lyso-PE-18:1 met the additional criterion of having a fold change ≥ 1.5 or ≤ –1.5. Furthermore, the rich growth medium used in this study may have left some anabolic pathways inactivated, potentially excluding a group of metabolites that correlate with the virulence phenotype under different growth conditions.

In contrast to the primary metabolome, the secondary metabolome was substantially different between the two groups. The variance between the secondary metabolomes of the two groups enabled a group separation based solely on the abundances of identified virulence-associated secondary metabolites (Figure 4). The ability to detect all major groups of virulence-associated secondary metabolites in *P. aeruginosa* in a single LC-MS method underlines the usefulness of this technology in gauging pseudomonal virulence and its relation to metabolism. The strong upregulation of virulence-associated metabolites is responsible for the asymmetry in the volcano plot (Figure S3), that exhibits a substantially larger number of upregulated features compared to downregulated features. The most important differentially abundant secondary metabolites discovered were AQs, which are known to regulate virulence in several ways [23]. In a recent study using Rapid Evaporative Ionisation Mass Spectrometry to differentiate CF and non-CF *P. aeruginosa* isolates, Bardin et al. identified AQs and rhamnolipids as important features for phenotype classification, with lower AQ levels in mucoid isolates [65]; thereby highlighting the notion that AQ profiles are an integral part of strain-specific metabolic profiles in *P. aeruginosa*. The abundance of quorum sensing signaling molecules in clinical isolates have been investigated in depth. Despite their role in the regulation of virulence, low to non-existant levels have been observed in infectious clinical isolates [66], correlating with the low AQ levels measured in our avirulent clinical strains. Furthermore, Davenport et al. demonstrated that roughly 1/3 of the *P. aeruginosa* metabolome is linked to quorum sensing, with primary and secondary metabolite levels affected by lack of quorum sensing signaling molecules [67]. Accordingly, AQs are among the most relevant features of our random forest classification model that successfully differentiates between virulent and avirulent strains.

Recent research on the CF sputum microbiome and metabolome highlights the importance of AQs, rhamnolipids and phenazines in the *in vivo* virulence in the human host and suggests a correlation of the abundance of these secondary metabolites and clinical disease severity [68,69]. In line with findings by Quinn et al., the prominent AQ PQS was not among the most important metabolites associated with infection and virulence [68], whereas a rhamnolipid (Rha-Rha-C10-C10 in their case) was [69]. The fact that the same molecules we identified as virulence-associated in our cultivation-based approach or closely related ones are also connected to virulence in a clinical human setting further supports the validity of our findings.

Furthermore, our search for discriminatory markers of virulence and biofilm phenotypes pointed towards several features that could not be identified. Our random forest classification model included seven ‘unknowns’ in the group of the 10 most important features. These were within a broad *m/z* range from 85 Da to 464 Da, suggesting that they belong to different metabolite classes (Figure S17). The most important feature in our model is an ‘unknown’ feature that clearly separates the two groups in our discovery data set and performs acceptably in our validation data set. Its putative identity is speculative; a sum formula of C_12_H_15_N_2_ for the cation points towards a phenazine-like structure, and the correlation with other features suggests a link to AQ biosynthesis. However, caution needs to be taken in the interpretation of unknowns in LC-MS metabolomics, as they may actually derive from other metabolites or represent artifacts [70,71] and AQ and phenazine levels are of course not independent of each other due to multiple direct and indirect regulatory effects [23]. The fact that an unknown *P. aeruginosa* metabolite is a putative virulence biomarker demonstrates the value of studying the secondary metabolites of pathogenic bacteria, including those produced by highly studied species. Further efforts are needed to elucidate the structure of the feature in question and study its role in pseudomonal virulence.

As with any *in vitro* model, the work presented here comes with limitations. Quinn et al. have shown that significant differences exist between cultured *P. aeruginosa* strains and those in the host environment [68]. Important factors such as interspecies relations [72], and the duration of infection before the sampling and isolation of the strains [73] were outside the scope of this study. The virulence assessment in *G. mellonella* presents an additional limitation as it might not fully reflect virulence properties in the human host. Differences in metabolite diversity as measured by the Shannon index have been observed, but the current dataset is insufficient to conclude whether and how strain-specific metabolite diversity is related to the virulence phenotype; this aspect remains to be investigated in future studies.

However, the combination of known metabolites and unannotated features in a random forest classification model achieves an area under the ROC curve of > 0.8 for the validation data set, achieving a good discrimination of virulent and avirulent *P. aeruginosa* strains. The model is, at present, not suitable for virulence prediction of strains with different biofilm morphologies. The inference of virulence phenotypes of *P. aeruginosa* clinical strains cannot be achieved from genomics data alone [14], and are difficult to construe from transcriptomics data [18,19]. Thus, the strength of LC-MS metabolomics in classifying *P. aeruginosa* strains is a logical reflection of the high relevance of secondary metabolites for infection processes in this pathogen.

## 5. Conclusions

*P. aeruginosa* clinical strains with different virulence and biofilm phenotypes have different metabolic profiles. A large portion of these differences can be attributed to known virulence-associated secondary metabolites; however, structurally unidentified features are important separators and putative virulence biomarkers. Using machine learning on the complete metabolome dataset, we obtained a classification model that differentiates virulent and avirulent *P. aeruginosa* clinical strains with good accuracy (area under the ROC curve of > 0.8). We have demonstrated that metabolomics can play an important role in the discovery of reliable virulence biomarkers or biomarker panels that are applicable in the clinic to gauge virulence and provide invaluable information on the potential clinical course of an infection.

## Figures and Tables

**Table 1:** Strains used in this study. Strains are listed by their identifier in the Bactome data base [35]. The biofilm cluster corresponds to the biofilm morphology [15] and the survival rate in a *Galleria mellonella* infection model (according to [34]) is given in percent survival after 48 h. The infection or sampling site is indicated if available. nd—not determined.

**Discovery Data Set**			
**Strain**	**Biofilm Cluster**	***Galleria*** **Survival (48 h)**	**Infection/Sampling Site**
CH2860	A	5	Respiratory tract
CH4528	A	0	Respiratory tract
ESP046	A	0	nd/other
ESP088	A	5	nd/other
F2030	A	0	Respiratory tract
MHH16798	A	20	Respiratory tract
ZG302383	A	0	nd/other
CH2682	B	95	Rectal swab
ESP027	B	100	nd/other
F1959	B	100	Respiratory tract
F2165	B	100	Respiratory tract
F2166	B	100	Respiratory tract
F2224	B	95	nd/other
MHH17767	B	100	Respiratory tract
**Validation Data Set**			
**Strain**	**Biofilm Cluster**	***Galleria*** **Survival (48 h)**	**Infection/Sampling Site**
CH2690	A	0	Rectal swab
ESP058	A	0	nd/other
ESP067	A	5	nd/other
F1997	A	0	Rectal swab
MHH17704	A	5	nd/other
Psae1439	A	10	Respiratory tract
ZG8038581181	A	10	Respiratory tract
CH4681	B	90	Respiratory tract
F1764	B	95	Respiratory tract
F2020	B	95	Wound infection
MHH16050	B	60	nd/other
MHH16563	B	95	Respiratory tract
MHH17546	B	100	Respiratory tract
Psae1837	B	75	nd/other
**Additional Data Set**			
**Strain**	**Biofilm Cluster**	***Galleria*** **Survival (48 h)**	**Infection/Sampling Site**
CH2706	C	0	Rectal swab
CH4591	C	0	Rectal swab
ESP083	C	0	nd/other
F1864	C	0	nd/other
F2059	C	0	Wound infection
ZG316717	C	5	Ear infection
ZG8510487	C	0	Urinary tract infection

## Supplementary Materials

The following are available online at https://www.mdpi.com/2218-273X/10/7/1041/s1, Figure S1: PCA scores plot of the discovery data set, Figure S2: Volcano plot of the discovery data set, Figure S3: PCA scores plot with only annotated features considered in the analysis, Figure S4: PCA loadings plot of the discovery data set, Figure S5: Transcriptional profiles reveal no gene expression pattern associated with the virulence phenotype, Figure S6: Levels of the two phenazines pyocyanin and phenazine-1-carboxylic acid, in the different strains of the discovery data set, Figure S7: Rhamnolipid levels in the different strains of the discovery data set, Figure S8: Structures of annotated rhamnolipids, Figure S9: Credentialed peak pair of M187T6_2 and its ^13^C-labeled derivative, Figure S10: Full scan and MS^2^spectrum of the feaure M187T6_2 in the discovery data set, Figure S11: Pearson’s correlation of selected feature intensities to those of M187T6_2, Figure S12: Boxplots of feature intensities for M187T6_2 in the discovery and validation data set, Figure S13: PCA scores plot of the validation data set, Figure S14: Area under the ROC curve for a logistic regression model using the feature intensity of M187T6_2 to discriminate virulence phenotypes in the validation data set, Figure S15: Intra-group correlation of M187T6_2 with 48 h survival in the *Galleria mellonella* assay, Figure S16: Multidimensional scaling plot visualizing tree distances between the samples of the discovery data set, Figure S17: Variable importance plot displaying mean decrease in accuracy and mean decrease in impurity (Gini impurity) of the random forest model constructed from the discovery data set, Figure S18: Percentage of correctly predicted virulence phenotype in the validation set if run 100 times independently, Figure S19: PCA scores plot of the cluster C data set, Figure S20: Percentage of correctly predicted virulence phenotype in the validation set if run 100 times independently, Table S1: Harvesting data for discovery batch, Table S2: Harvesting data for the validation batch, Table S3: Harvesting data for the additional batch, Table S4: Metabolite identifications, Table S5: XCMS online parameters, Table S6: Transcriptomic fold changes of proteins associated with phenazine production, Table S7: Transcriptomic fold changes of proteins associated with pyochelin, rhamnolipid and alkylquinolone production, Table S8: Primary metabolites identified in this study. Metabolomics data have been deposited to the EMBL-EBI MetaboLights database (DOI:10.1093/nar/gkz1019, PMID:31691833) with the identifier MTBLS1749. The complete dataset can be accessed here https://www.ebi.ac.uk/metabolights/MTBLS1749.

## Abbreviations

The following abbreviations are used in this manuscript:

AQ	alkyl quinolone
AUC	area under the curve
CF	cystic fibrosis
DHQ	2,4-dihydroxyquinoline
ESI-QTOF-MS	electrospray ionisation quadrupole time-of-flight mass spectrometry
GNPS	Global Natural Product Social Molecular Networking
HSL	homeserine lactone
LC-MS	liquid chromatography–mass spectrometry
MASST	Mass Spectrometry Search Tool
*m/z*	mass-to-charge ratio
nd	not determined
nrpg	normalized reads per gene
OD_600_	optical density at 600 nm
p_adj_	adjusted *p*-value
PCA	principal component analysis
PE	phosphatidylethanolamine
PERMANOVA	Permutational multivariate analysis of variation
PQS	*Pseudomonas* quinolone signal
QNO	quinoline-*N*-oxide
Rha	rhamnose, rhamnosyl
ROC	receiver operating characteristics
VIP	variable importance in projection
