# Supplementary material for: Untargeted LC-MS Metabolomics Differentiates Between Virulent and Avirulent Clinical Strains of Pseudomonas aeruginosa
Source: Biomolecules. 2020 Jul 13;10(7):1041. doi: 10.3390/biom10071041 (PMC7407980; doi:10.3390/biom10071041)
Supplement: Supplementary file 1 [file biomolecules-10-01041-s001.pdf]

Supplementary Materials: Untargeted LC-MS Metabolomics Differentiates Between Virulent and Avirulent Clinical Strains of *Pseudomonas aeruginosa*

Tobias Depke, Janne Gesine Thöming, Adrian Kordes, Susanne Häussler and Mark Brönstrup

Supplementary Figures

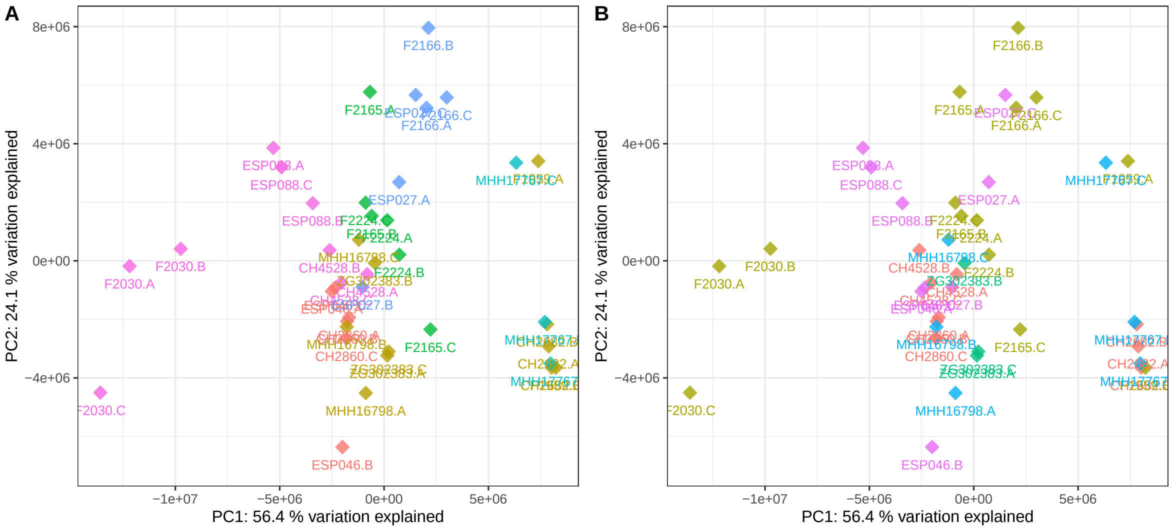

Figure S1. PCA scores plot of the discovery data set. Data points are coloured according to A) timepoint of harvest, i.e. duration of cultivation (red, 4 h; brown, 4.5 h; green, 5 h; cyan, 5.5 h; blue, 6 h; pink, 6.5 h), and B) geographical origin of the sample (red, Berlin (Germany); brown, Frankfurt am Main (Germany); green, Görlitz (Germany); blue, Hannover (Germany); pink, Palma de Mallorca (Spain)). No separation or grouping according to timepoint of harvest or geographical origin of the sample can be observed.

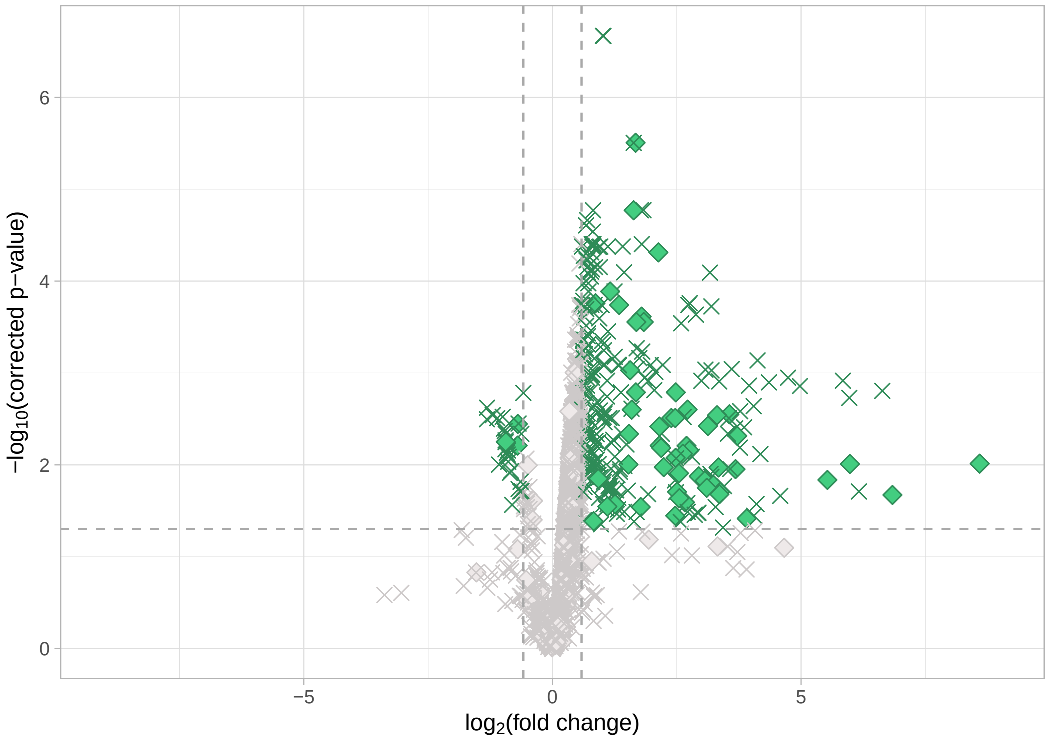

Figure S2. Volcano plot of the discovery data set. All features were plotted with the binary logarithm of their (non-directional) fold change on the x-axis and the negative decadic logarithm of their corrected p-value on the y-axis. Thresholds for significantly differentially abundant features are indicated by dashed lines (fold change ≥ 1.5, corrected p-value ≤ 0.05) and data points were colour coded according to these thresholds (green – significantly differentially abundant features, grey – other features). Diamonds signify identified features whereas unknowns are indicated by crosses. It is apparent that the majority of significantly differentially abundant features have higher levels in the virulent cluster A group which is consistent with the high number of virulence-associated secondary metabolites in the data set.

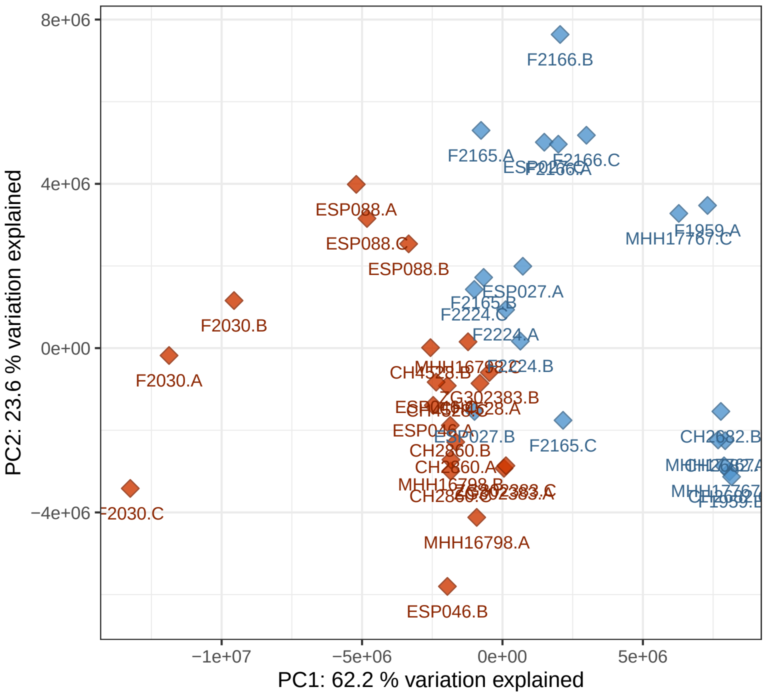

Figure S3. PCA scores plot with only annotated features considered in the analysis. The plot was generated analogously to Figure 1 in the main text. It suggests that the overall group separation is maintained if unknown features are ignored, indicating that the main drivers of separation or features correlated to them have been annotated. Red – virulent cluster A strains, blue – avirulent cluster B strains.

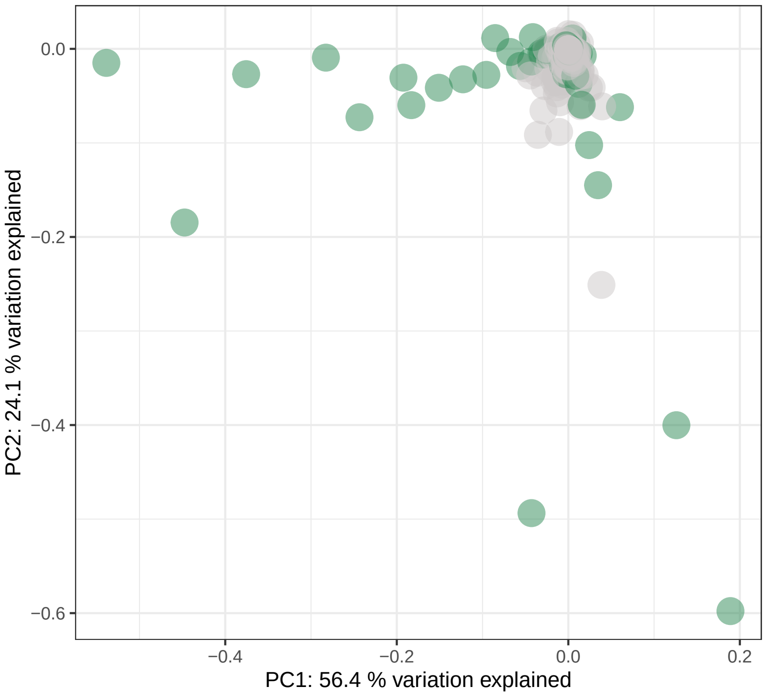

Figure S4. PCA loadings plot of the discovery data set. Green points symbolize annotated features and grey points features that could not be annotated. Most features with high loadings, i.e. a strong contribution to the first two principal components, have been annotated.

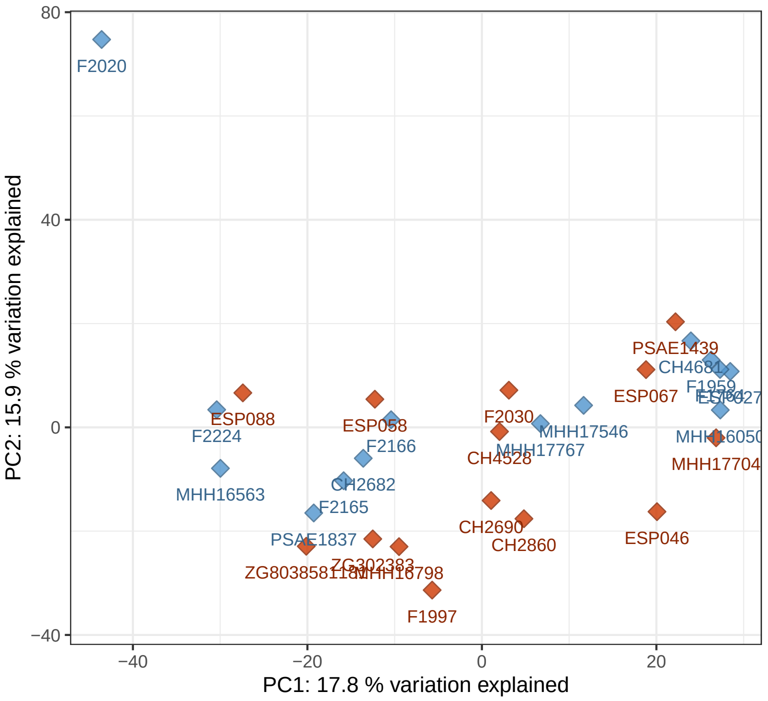

Figure S5. Transcriptional profiles reveal no gene expression pattern associated with the virulence phenotype. The principal component analysis (PCA) plot of transcriptional profiles recorded for clinical isolates grown under planktonic conditions does not cluster according to the observed *in vivo* virulence phenotype in the *G. mellonella* infection model. Each data point represents the transcriptional profile of an individual clinical isolate. Red – virulent cluster A strains, blue – avirulent cluster B strains.

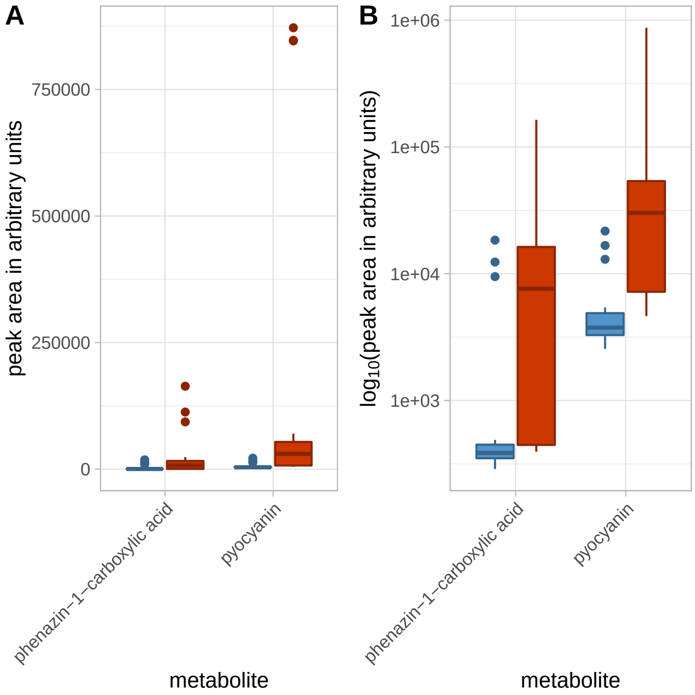

Figure S6. Levels of the two phenazines pyocyanin and phenazine-1-carboxylic acid, in the different strains of the discovery data set. Box plots of the peak areas in arbitrary units in the two phenotypic groups (A). A section of the y-scale with logarithmic scaling is shown to better visualize group differences (B). Both phenazine-1-carboxylic acid and pyocyanin have higher levels in the virulent strains, although there is significant overlap and both groups harbor one high producer strain each. Red – virulent cluster A strains, blue – avirulent cluster B strains.

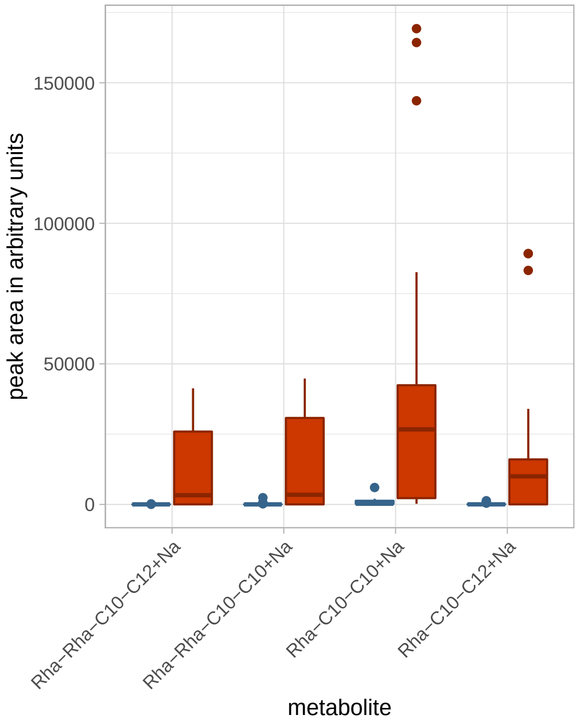

Figure S7. Rhamnolipid levels in the different strains of the discovery data set. Box plots of the peak areas of four annotated rhamnolipids in arbitrary units in the two phenotypic groups. Red – virulent cluster A strains, blue – avirulent cluster B strains.

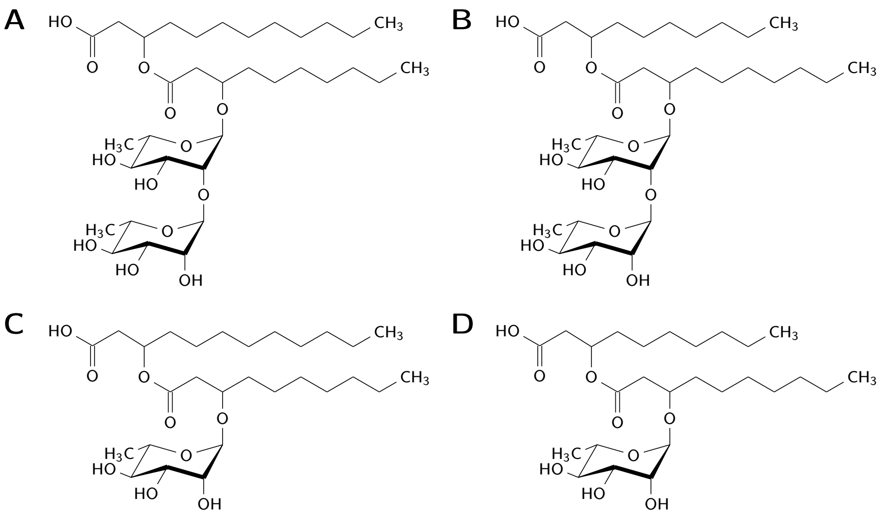

Figure S8. Structures of annotated rhamnolipids (cf. Figure S7). A: Rha-Rha-C10-C12. B: Rha-Rha-C10-C10. C: Rha-C10-C12. D: Rha-C10-C10.

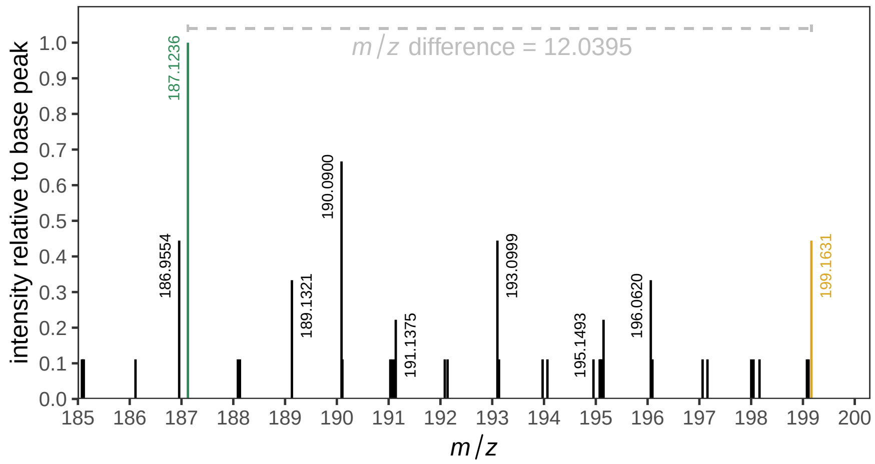

Figure S9. Credentialed peak pair of M187T6_2 and its ^{13}C-labeled derivative. Magnified section of a full scan MS spectrum with the peak corresponding to M187T6_2 marked in green and the one corresponding to its ^{13}C-labeled derivative marked in yellow. The measured *m/z* difference of 12.0395 is in accordance with the expected *m/z* difference between $C_{12}H_{15}N_2^+$ (the assumed formula of M187T6_2) and $^{13}C_{12}H_{15}N_2^+$ (theoretical *m/z* difference 12.0403). The incorporation of stable isotope labeled carbon from $^{13}C_6$-glucose in the growth medium provides evidence that the feature is a metabolite of biological origin rather than an artifact. The figure was adapted from [1].

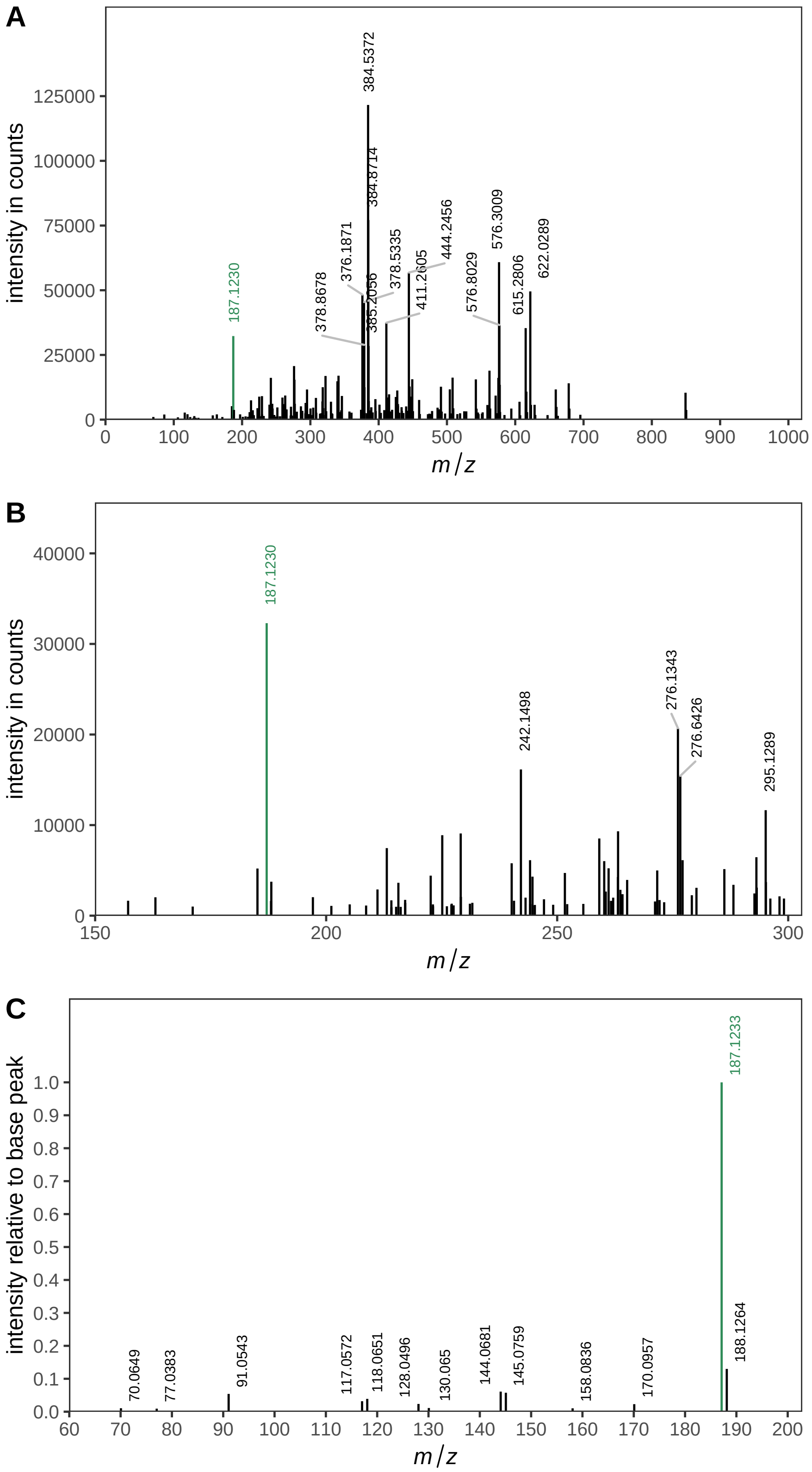

Figure S10. Full scan and MS^2 spectrum of the feaure M187T6_2 in the discovery data set. A: Full scan MS spectrum of the full *m/z* range from 0 to 1000. B: Same as A magnified to the relevant *m/z* range from 150 to 300. C: MS^2 spectrum of the 187.123 ion of M187T6_2. Peaks for the 187.123 ion are marked in green. The M187T6_2 feature displays a low abundance and its MS^2 spectrum is rather uninformative as the ion hardly shows fragmentation.

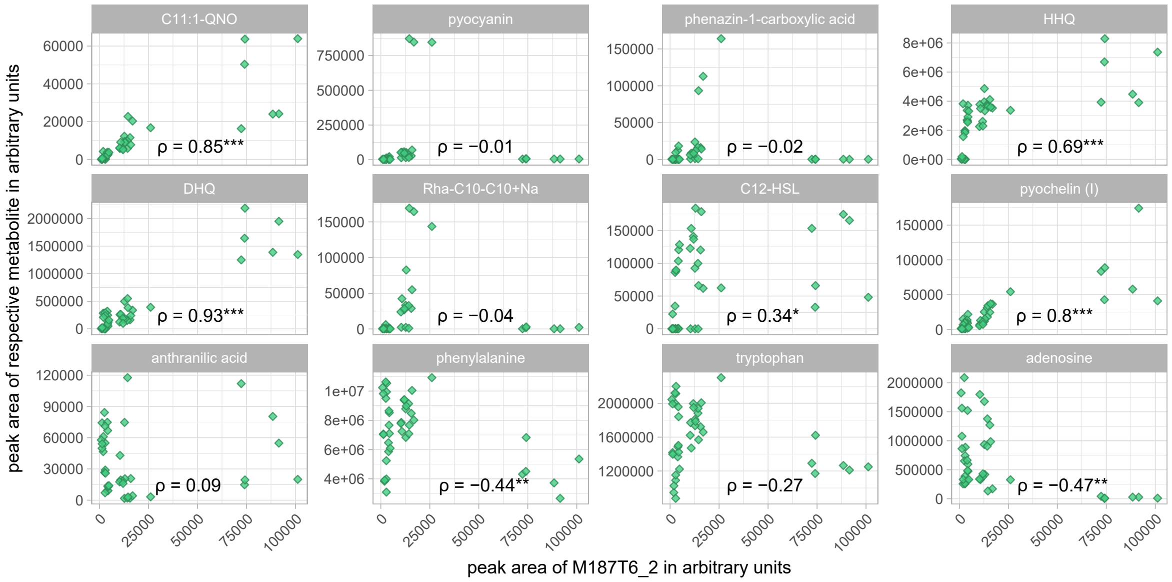

Figure S11. Pearson's correlation of selected feature intensities to those of M187T6_2. The peak area in arbitrary units of the feature M187T6_2 is plotted on the x-axis and the peak area of the respective metabolite in the sub-diagram title on the y-axis. Each data point corresponds to a biological replicate of a strain in the discovery data set. Pearson's correlation coefficient between the two respective peak areas is inserted as text in each sub-diagram with asterisks denoting statistical significance of the correlation (***, p-value ≤ 0.001; **, p-value ≤ 0.01; *, p-value ≤ 0.05; no asterisk, p-value > 0.05). Significant and strongly positive correlations can be found with AQs and the related DHQ as well as with pyochelin.

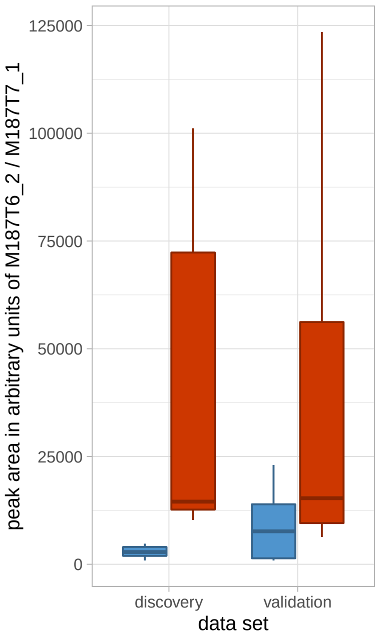

Figure S12. Boxplots of feature intensities for M187T6_2 in the discovery and validation data set. Due to automatic naming of the features during preprocessing by XCMS online, the respective feature has the identifier M187T7_1 in the validation data set. Peak areas in arbitrary units are used as a metric for the metabolite levels. While M187T6_2 is a perfect separator in the discovery data set, there is some overlap in the validation data set, i.e., the highest levels in the avirulent group exceed the lowest levels in the virulent group. The abundances in virulent cluster A and avirulent cluster B strains are significantly different. Red – virulent cluster A strains, blue – avirulent cluster B strains.

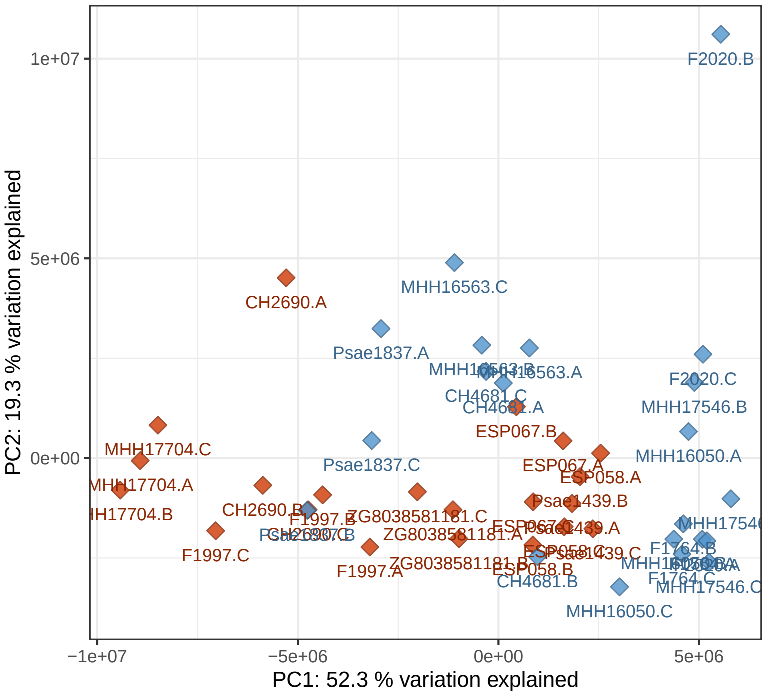

Figure S13. PCA scores plot of the validation data set. The plot was generated analogously to Figure 1 in the main text. Group separation appears to be weaker in the validation data set but is still possible. Red – virulent cluster A strains, blue – avirulent cluster B strains.

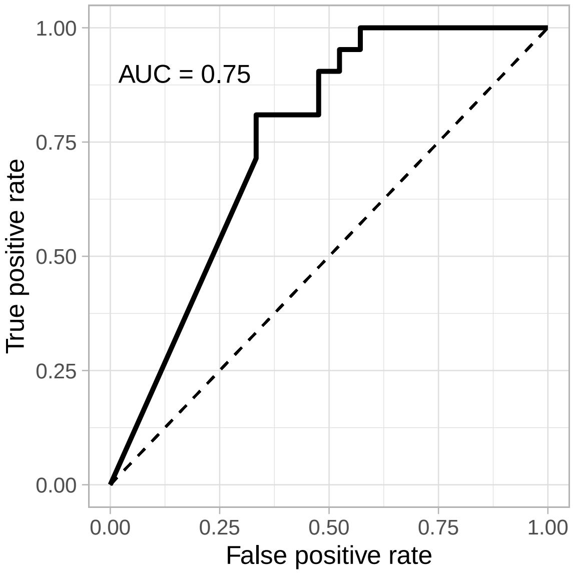

Figure S14. Area under the ROC curve for a logistic regression model using the feature intensity of M187T6_2 to discriminate virulence phenotypes in the validation data set. The Receiver Operating Characteristics curve was generated analogously to Figure 5 in the main text. An AUC of 0.75 signifies a decent classification performance, but is not sufficient for reliable differentiation of the phenotypes.

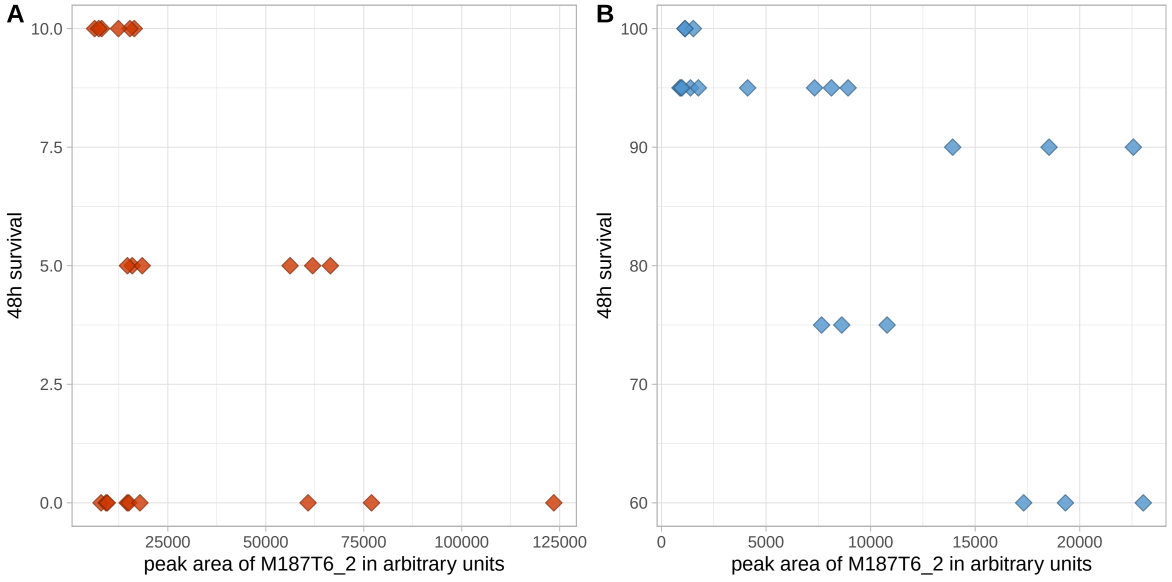

Figure S15. Intra-group correlation of M187T6_2 with 48h survival in the *Galleria mellonella* assay. The peak area in arbitrary units of M187T6_2 was plotted against the 48h survival in the *Galleria mellonella* assay. No clear correlation between the abundance of the candidate marker and the extent of virulence in the model could be identified. Red – virulent cluster A strains, blue – avirulent cluster B strains.

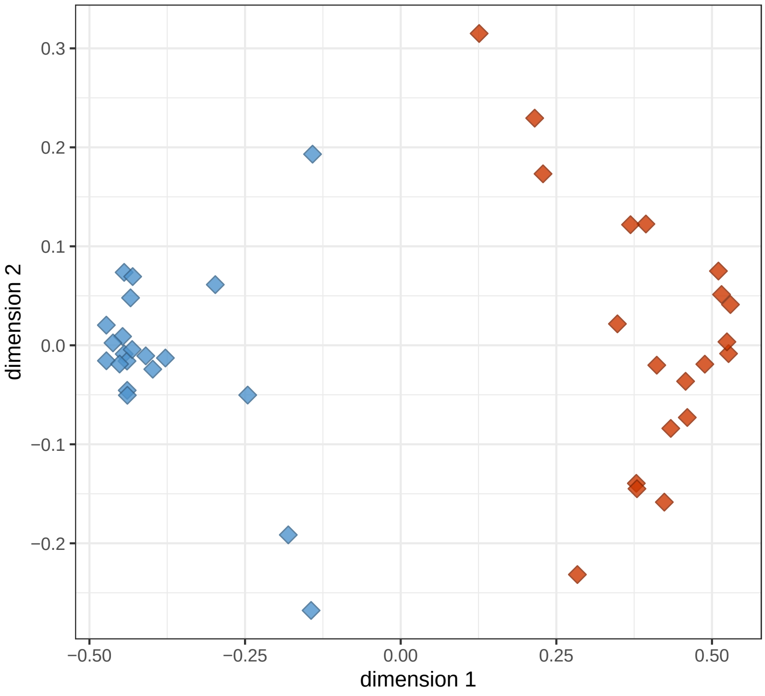

Figure S16. Multidimensional scaling plot visualizing tree distances between the samples of the discovery data set. Red – virulent cluster A strains, blue – avirulent cluster B strains.

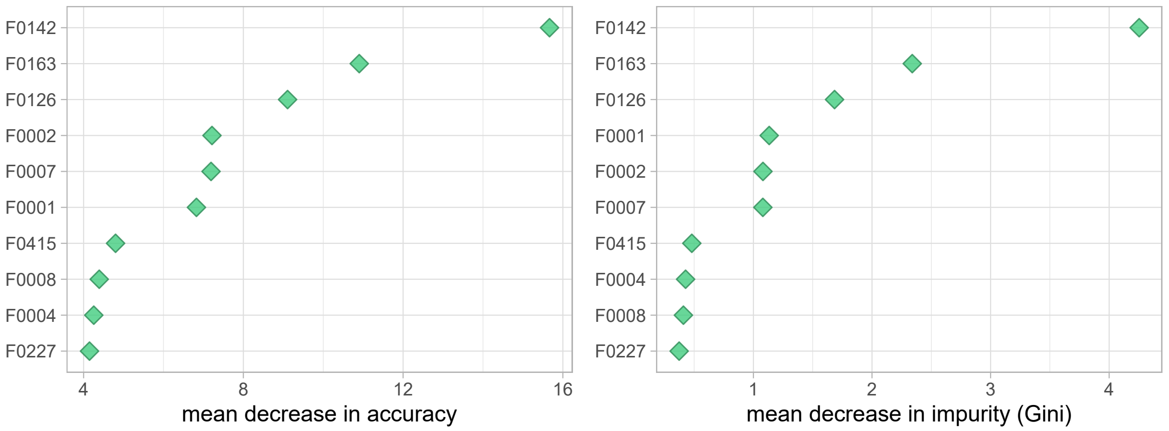

Figure S17. Variable importance plot displaying mean decrease in accuracy and mean decrease in impurity (Gini impurity) of the Random Forest model constructed from the discovery data set. The ten most important features are shown (identifiers are from the discovery data set): F0142 = M187T6_2, F0163 = M231T7_3, F0126 = C9-QNO, F0002 = M85T1_1, F0007 = C9:1-HQ, F0001 = M126T1_1, F0415 = M464T9_3, F0008 = C9:1-HQ , F0004 = M246T3_1, F0227 = M228T12.

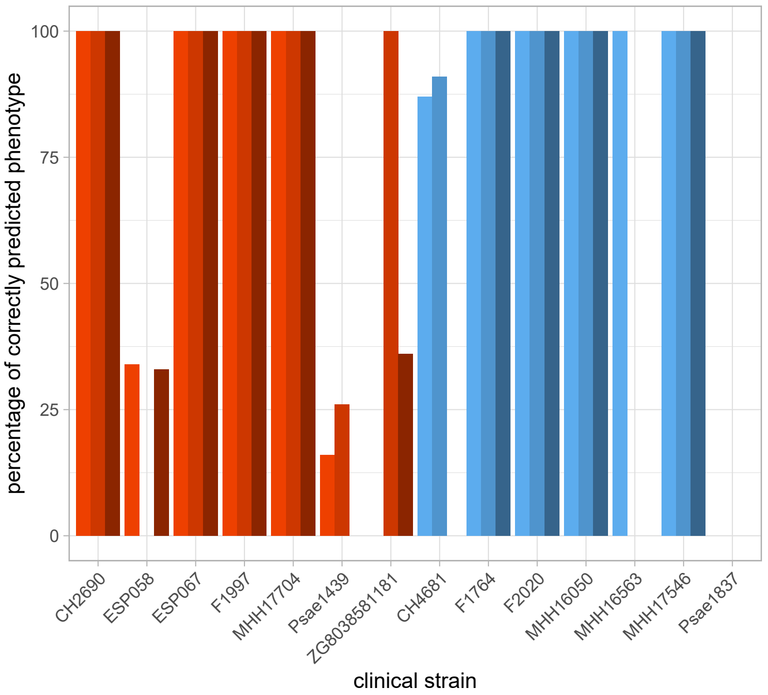

Figure S18. Percentage of correctly predicted virulence phenotype in the validation set if run 100 times independently. While eight strains are reliably assigned to the correct phenotype, three strains appear to be systematically misclassified. Red – virulent cluster A strains, blue – avirulent cluster B strains.

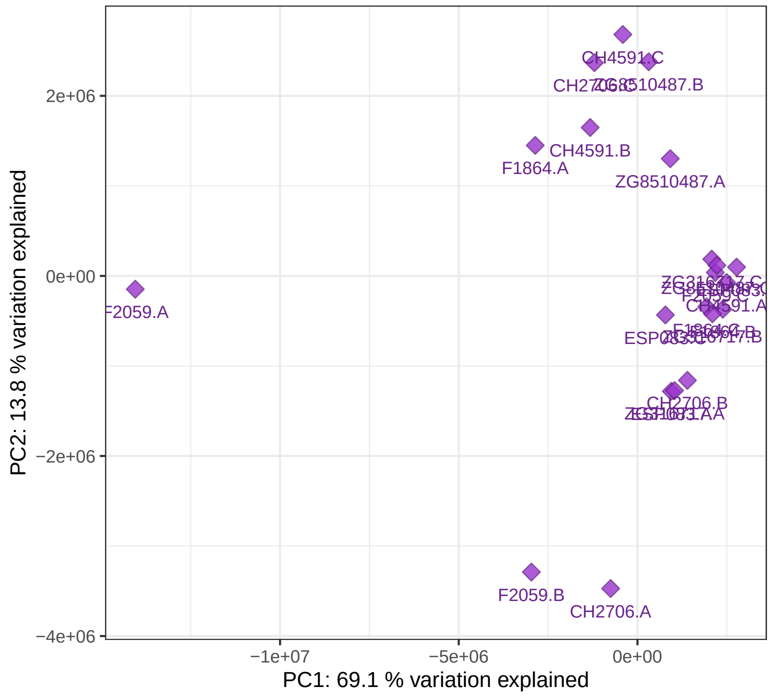

Figure S19. PCA scores plot of the Cluster 4 data set. The plot was generated analogously to Figure 1 in the main text.

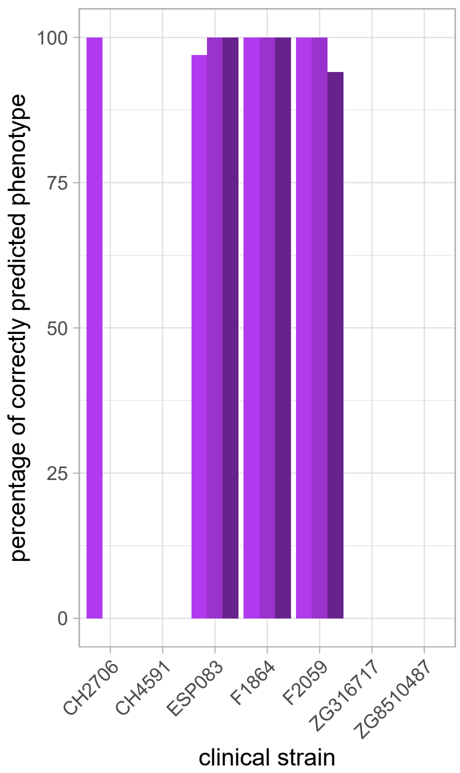

Figure S20. Percentage of correctly predicted virulence phenotype in the validation set if run 100 times independently (cf. Figure S18). Only three out of seven strains are reliably assigned to the correct virulence phenotype if the biofilm phenotype differs from those in the discovery data set.

Supplementary Tables

Table S1. Harvesting data for discovery batch. Each strain was cultivated in three biological replicates and the biomass of all three replicates was harvested at an OD_{600} of approximately 2. The exact OD_{600} and the timepoint of harvesting in hours after the start of cultivation is for each strain and replicate.

strain	OD_{600} at harvesting for individual replicates A	B	C	timepoint/h
CH2860	2.22	2.16	2.12	4.0
CH4528	1.71	1.76	1.52	6.5
ESP046	2.08	2.00	2.29	4.0
ESP088	2.01	1.88	1.84	6.5
F2030	2.05	2.08	1.99	6.5
MHH16798	2.01	1.84	2.10	4.5
ZG302383	1.95	1.99	1.94	4.5
CH2682	2.00	2.07	2.18	4.5
ESP027	2.42	2.23	2.33	6.0
F1959	2.10	1.85	1.98	4.5
F2165	2.85	2.16	2.39	5.0
F2166	2.04	2.50	2.58	6.0
F2224	2.33	2.32	2.25	5.0
MHH17767	1.68	1.77	2.45	5.5

Table S2. Harvesting data for the validation batch. The table is analogous to Table S1.

strain	OD_{600} at harvesting for individual replicates A	B	C	timepoint/h
CH2690	1.88	1.73	1.74	5.5
ESP058	1.90	2.08	1.84	6.5
ESP067	1.83	2.01	1.87	5.5
F1997	1.99	1.98	1.78	6.5
MHH17704	1.88	1.86	2.05	6.0
Psae1439	1.91	1.70	1.90	5.0
ZG8038581181	1.93	2.02	1.84	5.5
CH4681	2.13	2.00	1.87	5.0
F1764	2.05	1.85	2.07	6.5
F2020	2.50	1.28	1.46	5.0
MHH16050	2.03	2.00	2.40	6.0
MHH16563	1.64	1.58	1.60	7.5
MHH17546	2.22	1.92	1.96	7.0
Psae1837	1.82	1.95	1.69	5.5

Table S3. Harvesting data for the additional batch. The table is analogous to Table S1.

strain	OD_{600} at harvesting for individual replicates A	B	C	timepoint/h
CH2706	1.89	2.07	2.33	4.0
CH4591	1.92	1.98	2.56	4.0
ESP083	1.79	2.06	1.69	4.0
F1864	1.60	2.12	1.94	4.5
F2059	0.85	1.22	2.30	4.5
ZG316717	1.73	2.04	2.11	5.5
ZG8510487	2.40	2.36	2.49	4.5

Table S4. Metabolite identifications. Annotated features in the discovery data set as identified by their median m/z and median retention time. All annotations are assigned to a metabolite or metabolite class. The identification level is given according to the Metabolomics Standards Initiative [2]: 1, identified by comparison of at least two orthogonal characteristics to an authentic standard; 2, annotated as a distinct compound by comparison with a compound database or the scientific literature; 3, annotated as member of a distinct compound class. The last column states by the use of which properties the compound was identified/annotated: RT, retention time; MS, full scan MS spectrum (exact *m/z*, (in-source) fragmentation, isotopic pattern); MSMS, tandem MS fragmentation pattern.

Identifier in the discovery data set	Median *m/z*	Median retention time [min]	Annotation	Metabolite	Comment	Identification Level	Identified by
M112T1_4	112.1118	0.96	spermidine (fragment) (I)	spermidine	(in-source) fragment	1	RT, MS, MSMS
M129T1_4	129.1385	0.96	spermidine (fragment) (II)	spermidine	(in-source) fragment	1	RT, MS, MSMS
M146T1_5	146.1651	0.98	spermidine	spermidine		1	RT, MS, MSMS
M89T1	89.1071	0.98	putrescine	putrescine		1	RT, MS
M175T1_6	175.1190	1.09	arginine	arginine		1	RT, MS, MSMS
M156T1_5	156.0766	1.10	histidine	histidine		1	RT, MS
M130T1_4	130.0497	1.12	5-oxoproline (I)	5-oxoproline		1	RT, MS, MSMS
M104T1_3	104.1070	1.12	choline	choline		1	RT, MS, MSMS
M148T1_2	148.0604	1.13	glutamic acid	glutamic acid		1	RT, MS, MSMS
M176T1_2	176.1031	1.14	citrullline	citrullline		1	RT, MS, MSMS
M365T1_4	365.1057	1.14	sugar	undetermined sugar	various possibilities	3	MS, MSMS
M191T1_5	191.1017	1.18	2,6-diaminoheptanedioic acid	2,6-diaminoheptanedioic acid		1	RT, MS
M116T1_3	116.0705	1.18	proline (I)	proline		1	RT, MS
M219T1_4	219.0975	1.19	Glu Ala	Glu Ala		2	MS, MSMS
M147T1_3	147.1126	1.26	lysine	lysine		1	RT, MS
M146T1_3	146.0921	1.34	4-guanidinobutyric acid	4-guanidinobutyric acid		1	RT, MS
M535T1_2	535.1880	1.34	Glu Glu Glu Glu (I)	Glu Glu Glu Glu		2	MS, MSMS
M106T1	106.0489	1.34	serine	serine		1	RT, MS
M124T1_2	124.0391	1.34	nicotinic acid (I)	nicotinic acid		1	RT, MS
M136T1_3	136.0615	1.34	adenine	adenine		1	RT, MS
M308T1_2	308.0906	1.35	glutathione	glutathione	double positive charge	1	RT, MS
M130T1_5	130.0499	1.36	5-oxoproline (II)	5-oxoproline		1	RT, MS, MSMS
M123T1_3	123.0550	1.36	nicotinamide (I)	nicotinamide		2	MS, MSMS

Identifier in the discovery data set	Median *m/z*	Median retention time [min]	Annotation	Metabolite	Comment	Identification Level	Identified by
M137T1_2	137.0456	1.37	hypoxanthine (I)	hypoxanthine		1	RT, MS
M124T2_1	124.0390	1.54	nicotinic acid (II)	nicotinic acid		1	RT, MS
M190T2_1	190.0707	1.59	N-acetylglutamate	N-acetylglutamate		2	MS, MSMS
M116T2	116.0703	1.61	proline (II)	proline		1	RT, MS
M123T2_2	123.0548	1.65	nicotinamide (II)	nicotinamide		2	MS, MSMS
M118T2	118.0860	1.66	betaine	betaine		1	RT, MS, MSMS
M130T2	130.0498	1.67	5-oxoproline (III)	5-oxoproline		1	RT, MS, MSMS
M169T2	169.0353	1.68	uric acid	uric acid		1	RT, MS
M333T2_1	332.5617	1.76	NAD (2+)	NAD	double positive charge	2	MS, MSMS
M153T2_2	153.0403	1.77	xanthine (I)	xanthine		1	RT, MS
M664T2	664.1162	1.77	NAD	NAD		2	MS, MSMS
M137T2_1	137.0456	1.79	hypoxanthine (II)	hypoxanthine		1	RT, MS
M535T2	535.1877	1.81	Glu Glu Glu Glu (II)	Glu Glu Glu Glu		2	MS, MSMS
M348T2_1	348.0699	1.88	adenosine-5′-monophosphate	adenosine-5′-monophosphate		1	RT, MS, MSMS
M132T2_2	132.1019	1.88	Leucine / Isoleucine / Norleucine	Leucine / Isoleucine / Norleucine	the three species could not be distinguished in the experimental setting	3	RT, MS, MSMS
M364T2	364.0649	2.02	guanosine-5′-monophosphate	guanosine-5′-monophosphate		1	RT, MS
M330T2_1	330.0595	2.03	adenosine-2′,3′-cyclic monophosphate	adenosine-2′,3′-cyclic monophosphate		1	RT, MS
M153T2_1	153.0404	2.04	xanthine (II)	xanthine		1	RT, MS
M164T2	164.0562	2.33	pterine	pterine		2	MS, MSMS
M268T3_1	268.1041	2.68	adenosine	adenosine		1	RT, MS, MSMS
M137T3_2	137.0456	2.74	hypoxanthine (III)	hypoxanthine		1	RT, MS
M140T3	140.0341	2.82	6-hydroxynicotinic acid	6-hydroxynicotinic acid		1	RT, MS
M182T3_2	182.0809	2.98	tyrosine	tyrosine		1	RT, MS, MSMS
M166T3_1	166.0862	3.46	phenylalanine	phenylalanine		1	RT, MS, MSMS
M120T3_2	120.0807	3.46	phenylalanine (fragment) (I)	phenylalanine	(in-source) fragment	1	RT, MS, MSMS

**Identifier in the discovery data set**	**Median *m/z***	**Median retention time [min]**	**Annotation**	**Metabolite**	**Comment**	**Identification Level**	**Identified by**
M103T3	103.0542	3.46	phenylalanine (fragment) (III)	phenylalanine	(in-source) fragment	1	RT, MS, MSMS
M219T5	219.1337	5.24	Ser Leu	Ser Leu		2	MS, MSMS
M220T5_2	220.1179	5.28	D-pantothenic acid	D-pantothenic acid		1	RT, MS, MSMS
M598T5_1	597.6777	5.44	UDP-muramyl-pentapeptide	UDP-muramyl-pentapeptide		2	MS, MSMS
M360T6_6	360.2127	5.68	Ile Val Glu / Val Ile Glu (I)	Ile Val Glu / Val Ile Glu	possibly structural isomer	3	MS, MSMS
M188T6_2	188.0707	5.93	tryptophan (fragment) (I)	tryptophan		1	RT, MS
M205T6_1	205.0972	5.93	tryptophan	tryptophan		1	RT, MS
M160T6_1	160.0754	5.93	indole-3-acetaldehyde	indole-3-acetaldehyde		1	RT, MS
M144T6_3	144.0807	5.93	tryptamine (I)	tryptamine		1	RT, MS
M298T6_4	298.0968	6.26	5′-methylthioadenosine	5′-methylthioadenosine		1	RT, MS, MSMS
M211T6_3	211.0865	6.30	pyocyanin	pyocyanin		1	RT, MS, MSMS
M188T6_1	188.0703	6.35	tryptophan (fragment) (II)	tryptophan		1	RT, MS
M360T6_7	360.2123	6.36	Ile Val Glu / Val Ile Glu (II)	Ile Val Glu / Val Ile Glu	possibly structural isomer	3	MS, MSMS
M378T7_3	378.2023	6.56	Pro Tyr Val	Pro Tyr Val		2	MS, MSMS
M295T7_4	295.1290	6.56	Glu Phe	Glu Phe		2	MS, MSMS
M144T7_1	144.0806	6.71	tryptamine (II)	tryptamine		1	RT, MS
M328T7_7	328.2233	6.81	Pro Leu Val (I)	Pro Leu Val		2	MS, MSMS
M263T7_2	263.1389	7.01	Pro Phe (I)	Pro Phe		2	MS, MSMS
M138T7	138.0548	7.09	anthranilic acid	anthranilic acid		1	RT, MS
M120T7	120.0444	7.09	anthranilic acid (fragment)	anthranilic acid	(in-source) fragment	1	RT, MS
M328T7_8	328.2233	7.24	Pro Leu Val (II)	Pro Leu Val		2	MS, MSMS
M263T7_3	263.1385	7.29	Pro Phe (II)	Pro Phe		2	MS, MSMS
M342T8_7	342.2389	7.79	Pro Ile Leu or isomer	Pro Ile Leu or isomer	possibly structural isomer	3	MS, MSMS
M344T8_10	344.2540	8.21	Leu Leu Val or isomer	Leu Leu Val or isomer	possibly structural isomer	3	MS, MSMS
M162T8	162.0550	8.35	DHQ	DHQ		1	RT, MS, MSMS

Identifier in the discovery data set	Median *m/z*	Median retention time [min]	Annotation	Metabolite	Comment	Identification Level	Identified by
M243T9_3	243.0875	9.19	lumichrome	lumichrome	possible riboflavin fragment	2	MS, MSMS
M216T11	216.1382	11.04	C5-HQ	C5-HQ		2	MS, MSMS
M232T11	232.1330	11.30	C5-QNO	C5-QNO		2	MS, MSMS
M225T11	225.0658	11.45	phenazin-1-carboxylic acid	phenazin-1-carboxylic acid		1	RT, MS, MSMS
M325T12_2	325.0674	11.69	pyochelin (I)	pyochelin		1	RT, MS, MSMS
M230T12	230.1537	12.10	C6-HQ	C6-HQ		2	MS, MSMS
M325T12_1	325.0672	12.34	pyochelin (II)	pyochelin		1	RT, MS, MSMS
M288T13	288.1959	12.66	C9-QNO (I)	C9-QNO		2	MS, MSMS
M258T13	258.1487	12.70	C7:1-QNO	C7:1-QNO		2	MS, MSMS
M242T13	242.1541	13.05	C7:1-HQ	C7:1-HQ		2	MS, MSMS
M244T13_1	244.1697	13.10	HHQ	HHQ		1	RT, MS, MSMS
M286T13	286.1798	13.11	C9:1-QNO (I)	C9:1-QNO		2	MS, MSMS
M159T13	159.0676	13.11	HHQ (fragment)	HHQ	(in-source) fragment	1	RT, MS, MSMS
M260T13	260.1647	13.18	C7-QNO	C7-QNO		1	RT, MS, MSMS
M314T14	314.2112	13.64	C11:1-QNO	C11:1-QNO		2	MS, MSMS
M256T14	256.1695	14.01	C8:1-HQ	C8:1-HQ		2	MS, MSMS
M258T14	258.1854	14.04	C8-HQ	C8-HQ		2	MS, MSMS
M274T14	274.1800	14.06	C8-QNO	C8-QNO		2	MS, MSMS
M286T14	286.1802	14.24	C9:1-QNO (II)	C9:1-QNO		2	MS, MSMS
M320T14	320.1833	14.28	C12-HSL	C12-HSL		1	RT, MS, MSMS
M270T14	270.1854	14.33	C9:1-HQ (I)	C9:1-HQ		2	MS, MSMS
M316T14	316.2268	14.40	C11-QNO	C11-QNO		2	MS, MSMS
M268T14	268.1694	14.43	C9:2-HQ	C9:2-HQ		2	MS, MSMS
M270T15_2	270.1854	14.57	C9:1-HQ (II)	C9:1-HQ		2	MS, MSMS
M300T15_1	300.1955	14.74	C10:1-QNO (I)	C10:1-QNO		2	MS, MSMS
M284T15	284.2008	14.86	C10:1-HQ (I)	C10:1-HQ		2	MS, MSMS
M288T15	288.1960	14.92	C9-QNO (II)	C9-QNO		2	MS, MSMS
M270T15_1	270.1855	14.94	C9:1-HQ (III)	C9:1-HQ		2	MS, MSMS
M272T15_2	272.2012	14.96	C9-HQ	C9-HQ		2	MS, MSMS
M300T15_2	300.1956	15.27	C10:1-QNO (II)	C10:1-QNO		2	MS, MSMS

Identifier in the discovery data set	Median *m/z*	Median retention time [min]	Annotation	Metabolite	Comment	Identification Level	Identified by
M296T16_2	296.2009	15.51	C11:2-HQ (I)	C11:2-HQ		2	MS, MSMS
M314T16_1	314.2116	15.57	C11:1-PQS (I)	C11:1-PQS		2	MS, MSMS
M298T16_1	298.2167	15.61	C11:1-HQ (I)	C11:1-HQ		2	MS, MSMS
M284T16	284.2009	15.67	C10:1-HQ (II)	C10:1-HQ		2	MS, MSMS
M302T16	302.2113	15.76	C10-QNO	C10-QNO		2	MS, MSMS
M296T16_1	296.2010	15.80	C11:2-HQ (II)	C11:2-HQ		2	MS, MSMS
M314T16_3	314.2113	15.84	C11:1-PQS (II)	C11:1-PQS		2	MS, MSMS
M286T16	286.2166	15.84	C10-HQ	C10-HQ		2	MS, MSMS
M298T16_2	298.2166	15.90	C11:1-HQ (II)	C11:1-HQ		2	MS, MSMS
M673T16	673.3766	15.91	Rha-Rha-C10-C10+Na	Rha-Rha-C10-C10	Na adduct	2	MS, MSMS
M454T16_2	454.2929	16.06	PE(16:0/0:0) (I)	PE(16:0/0:0)		1	RT, MS, MSMS
M314T16_2	314.2116	16.12	C11:1-PQS (III)	C11:1-PQS		2	MS, MSMS
M298T16_3	298.2166	16.32	C11:1-HQ (III)	C11:1-HQ		2	MS, MSMS
M328T16	328.2269	16.38	C12:1-QNO	C12:1-QNO		2	MS, MSMS
M454T16_1	454.2930	16.41	PE(16:0/0:0) (II)	PE(16:0/0:0)		1	RT, MS, MSMS
M312T16	312.2321	16.47	C12:1-HQ	C12:1-HQ		2	MS, MSMS
M316T17	316.2273	16.64	C11-PQS	C11-PQS		2	MS, MSMS
M298T17_1	298.2167	16.74	C11:1-HQ (IV)	C11:1-HQ		2	MS, MSMS
M300T17	300.2323	16.75	C11-HQ	C11-HQ		2	MS, MSMS
M527T17	527.3190	16.76	Rha-C10-C10+Na	Rha-C10-C10	Na adduct	2	MS, MSMS
M480T17	480.3087	16.76	PE(18:1/0:0)	PE(18:1/0:0)		1	RT, MS, MSMS
M502T17	502.2904	16.76	PE(18:1/0:0) +Na	PE(18:1/0:0)	Na adduct	1	RT, MS, MSMS
M342T17	342.2429	17.06	C13:1-PQS	C13:1-PQS		2	MS, MSMS
M326T17_1	326.2480	17.17	C13:1-HQ (I)	C13:1-HQ		2	MS, MSMS
M701T17	701.4080	17.29	Rha-Rha-C10-C12+Na	Rha-Rha-C10-C12	Na adduct	2	MS, MSMS
M326T17_2	326.2475	17.41	C13:1-HQ (II)	C13:1-HQ		2	MS, MSMS
M555T18	555.3506	18.17	Rha-C10-C12+Na	Rha-C10-C12	Na adduct	2	MS, MSMS
M326T18	326.2477	18.49	C13:1-HQ (III)	C13:1-HQ		2	MS, MSMS
M328T19	328.2635	18.51	C13-HQ	C13-HQ		2	MS, MSMS
M255T19	255.2316	18.60	palmitoleic acid	palmitoleic acid		1	RT, MS
M260T20	260.1646	19.93	PQS	PQS		1	MS, MSMS

Identifier in the discovery data set	Median *m/z*	Median retention time [min]	Annotation	Metabolite	Comment	Identification Level	Identified by
M327T20	327.2269	19.93	oleic acid	oleic acid		1	RT, MS

Table S5. XCMS online parameters. The LC-MS data set was (pre-)processed using XCMS online [3]. The parameters were chosen to fit the analytical machinery used to generate the data and partly modified on an empirical basis.

preprocessing step	parameter	value	explanation
feature detection	method	centWave	peak finding algorithm based on continuous wavelet transformation
	ppm	15	allowable *m/z* deviation in consecutive scans, expressed in parts per million
	minimum peak width	10.9	chromatographic peak widths in s
	maximum peak width	31.12	
	signal/noise threshold	10	minimum signal-to-noise ratio
	mzdiff	0.0155	minimum absolute *m/z* difference for overlapping chromatographic peaks
	integration method	1	based on Mexican hat filtered data
	prefilter peaks	3	minimum number of peaks with at least "prefilter intensity" to be retained after prefiltering
	prefilter intensity	100	minimum intensity of "prefilter peaks" (see above)
	noise filter	0	not necessary for centroided data
retention time correction	method	obiwarp	chromatographic alignment by "Ordered Bijective Interpolated Warping"
	profStep	1	*m/z* step size for profile generation
alignment	mzwid	0.026	*m/z* width of overlapping *m/z* slices used to group peaks across samples
	bw	5	maximum deviation of retention times in s
	minfrac	1	fraction of samples of one of the sample groups that have to display a group for it to be valid
	minsamp	1	number of samples of one of the sample groups that have to display a group for it to be valid
	max	50	upper threshold for the number of groups in one *m/z* slice
annotation	search for	isotopes + adducts	CAMERA considers both isotope peaks and possible adducts
	ppm	5	allowable relative *m/z* deviation between detected and expected peak, expressed in parts per million
	m/z absolute error	0.015	allowable absolute m/z deviation between detected and expected peak

Table S6. Transcriptomic fold changes of proteins associated with phenazine production. Most phenazine biosynthesis enzymes are significantly upregulated in virulent strains.

PA14 ID	Gene name	Product	$\log_2$(fold change)	adjusted p-value
PA14_09400	*phzS*	hypothetical protein	2.59	0.0170
PA14_09410	*phzG1*	pyrodoxamine 5′-phosphate oxidase	2.33	0.0274
PA14_09420	*phzF1*	phenazine biosynthesis protein	2.47	0.0216
PA14_09440	*phzE1*	phenazine biosynthesis protein PhzE	2.32	0.0338
PA14_09450	*phzD1*	phenazine biosynthesis protein PhzD	2.68	0.0503
PA14_09460	*phzC1*	phenazine biosynthesis protein PhzC	2.96	0.0105
PA14_09470	*phzB1*	phenazine biosynthesis protein	2.46	0.1104
PA14_09480	*phzA1*	phenazine biosynthesis protein	4.04	0.0001
PA14_09490	*phzM*	putative phenazine-specific methyltransferase	1.37	0.2548
PA14_39880	*phzG2*	pyridoxamine 5′-phosphate oxidase	2.33	0.0326
PA14_39890	*phzF2*	phenazine biosynthesis protein	2.56	0.0156
PA14_39910	*phzE2*	phenazine biosynthesis protein PhzE	2.36	0.0373
PA14_39925	*phzD2*	phenazine biosynthesis protein PhzD	2.31	0.0772
PA14_39945	*phzC2*	phenazine biosynthesis protein PhzC	2.68	0.0242
PA14_39960	*phzB2*	phenazine biosynthesis protein	3.22	0.0002
PA14_39970	*phzA2*	phenazine biosynthesis protein	3.47	0.0007

Table S7. Transcriptomic fold changes of proteins associated with pyochelin, rhamnolipid and alkylquinolone production. While corresponding metabolites are significantly more abundant in virulent strains, this difference is not reflected in the transcriptome data.

PA14 ID	Gene name	Product	$\log_2$(fold change)	adjusted p-value
PA14_09210	*pchA*	salicylate biosynthesis isochorismate synthase	1.08	0.3191
PA14_09220	*pchB*	isochorismate-pyruvate lyase	1.43	0.1719
PA14_09230	*pchC*	pyochelin biosynthetic protein PchC	1.31	0.2339
PA14_09240	*pchD*	pyochelin biosynthesis protein PchD	1.22	0.2412
PA14_09270	*pchE*	dihydroaeruginoic acid synthetase	1.50	0.1283
PA14_09280	*pchF*	pyochelin synthetase	1.24	0.2170
PA14_09290	*pchG*	pyochelin biosynthetic protein PchG	1.07	0.3105
PA14_09300	*pchH*	putative ATP-binding component of ABC transporter	1.16	0.2256
PA14_09320	*pchI*	putative ATP-binding component of ABC transporter	1.11	0.2530
PA14_09700	*pqsL*	putative monooxygenase	0.81	0.2270
PA14_19100	*rhlA*	rhamnosyltransferase chain A	1.54	0.2571
PA14_19110	*rhlB*	rhamnosyltransferase chain B	1.41	0.1992
PA14_19120	*rhlR*	transcriptional regulator RhlR	0.84	0.4892
PA14_19130	*rhlI*	autoinducer synthesis protein RhlI	0.79	0.6093
PA14_30630	*pqsH*	putative FAD-dependent monooxygenase	0.86	0.4270
PA14_49760	*rhlC*	rhamnosyltransferase 2	1.23	0.0833
PA14_51340	*mvfR*	Transcriptional regulator MvfR	0.55	0.2235
PA14_51350	*phnB*	anthranilate synthase component II	1.16	0.2558
PA14_51360	*phnA*	anthranilate synthase component I	1.45	0.1544
PA14_51380	*pqsE*	Quinolone signal response protein	1.10	0.3316
PA14_51390	*pqsD*	3-oxoacyl-(acyl carrier protein) synthase III	1.18	0.3168
PA14_51410	*pqsC*	PqsC	1.16	0.3356
PA14_51420	*pqsB*	PqsB	0.99	0.4767
PA14_51430	*pqsA*	coenzyme A ligase	1.37	0.2418

Table S8. Primary metabolites annotated in this study (cf. Table S4). Directional fold change and corrected p-value refer to the difference in abundance between virulent cluster A and avirulent cluster B isolates in the discovery data set.

Identifier in the discovery data set	Median *m/z*	Median retention time [min]	Directional fold change	Corrected p-value	Annotation	Metabolite
M191T1_5	191.1017	1.18	1.04	0.862	2,6-diaminoheptanedioic acid	2,6-diaminoheptanedioic acid
M146T1_3	146.0921	1.34	−1.26	0.423	4-guanidinobutyric acid	4-guanidinobutyric acid
M298T6_4	298.0968	6.26	−2.89	0.148	5′-methylthioadenosine	5′-methylthioadenosine
M130T1_4	130.0497	1.12	−1.11	0.643	5-oxoproline (I)	5-oxoproline
M130T1_5	130.0499	1.36	1.08	0.756	5-oxoproline (II)	5-oxoproline
M130T2	130.0498	1.67	1.12	0.646	5-oxoproline (III)	5-oxoproline
M140T3	140.0341	2.82	1.37	0.013	6-hydroxynicotinic acid	6-hydroxynicotinic acid
M136T1_3	136.0615	1.34	1.06	0.847	adenine	adenine
M268T3_1	268.1041	2.68	−1.41	0.297	adenosine	adenosine
M330T2_1	330.0595	2.03	1.23	0.040	adenosine-2′,3′-cyclic monophosphate	adenosine-2′,3′-cyclic monophosphate
M348T2_1	348.0699	1.88	−1.17	0.674	adenosine-5′-monophosphate	adenosine-5′-monophosphate
M138T7	138.0548	7.09	−1.33	0.390	anthranilic acid	anthranilic acid
M120T7	120.0444	7.09	−1.32	0.398	anthranilic acid (fragment)	anthranilic acid
M175T1_6	175.1190	1.09	1.21	0.578	arginine	arginine
M118T2	118.0860	1.66	1.19	0.206	betaine	betaine
M104T1_3	104.1070	1.12	−1.41	0.010	choline	choline
M176T1_2	176.1031	1.14	−1.44	0.179	citrullline	citrullline
M220T5_2	220.1179	5.28	1.28	0.009	D-pantothenic acid	D-pantothenic acid
M148T1_2	148.0604	1.13	−1.14	0.598	glutamic acid	glutamic acid
M308T1_2	308.0906	1.35	1.20	0.265	glutathione	glutathione
M364T2	364.0649	2.02	1.19	0.063	guanosine-5′-monophosphate	guanosine-5′-monophosphate
M156T1_5	156.0766	1.10	−1.06	0.516	histidine	histidine
M137T1_2	137.0456	1.37	1.52	0.125	hypoxanthine (I)	hypoxanthine
M137T2_1	137.0456	1.79	1.45	0.153	hypoxanthine (II)	hypoxanthine
M137T3_2	137.0456	2.74	1.15	0.840	hypoxanthine (III)	hypoxanthine
M160T6_1	160.0754	5.93	1.06	0.527	indole-3-acetaldehyde	indole-3-acetaldehyde

**Identifier in the discovery data set Metabolite**	**Median *m/z***	**Median retention time [min]**	**Directional fold change**	**Corrected p-value**	**Annotation**	**Metabolite**
M132T2_2	132.1019	1.88	1.20	0.041	Leucine / Isoleucine / Norleucine	Leucine / Isoleucine / Norleucine
M243T9_3	243.0875	9.19	1.27	0.003	lumichrome	lumichrome
M147T1_3	147.1126	1.26	1.16	0.098	lysine	lysine
M190T2_1	190.0707	1.59	−1.16	0.444	N-acetylglutamate	N-acetylglutamate
M664T2	664.1162	1.77	1.13	0.178	NAD	NAD
M333T2_1	332.5617	1.76	1.14	0.225	NAD (2+)	NAD (2+)
M123T1_3	123.0550	1.36	1.07	0.455	nicotinamide (I)	nicotinamide
M123T2_2	123.0548	1.65	1.12	0.495	nicotinamide (II)	nicotinamide
M124T1_2	124.0391	1.34	−1.13	0.735	nicotinic acid (I)	nicotinamide
M124T2_1	124.0390	1.54	−1.08	0.834	nicotinic acid (II)	nicotinamide
M327T20	327.2269	19.93	−1.32	0.040	oleic acid	oleic acid
M255T19	255.2316	18.60	−1.92	0.006	palmitoleic acid	palmitoleic acid
M454T16_2	454.2929	16.06	1.01	0.910	PE(16:0/0:0) (I)	PE(16:0/0:0)
M454T16_1	454.2930	16.41	−1.31	0.025	PE(16:0/0:0) (II)	PE(16:0/0:0)
M480T17	480.3087	16.76	−1.62	0.006	PE(18:1/0:0)	PE(18:1/0:0)
M502T17	502.2904	16.76	−1.62	0.004	PE(18:1/0:0) +Na	PE(18:1/0:0)
M166T3_1	166.0862	3.46	1.02	0.904	phenylalanine	phenylalanine
M120T3_2	120.0807	3.46	1.02	0.899	phenylalanine (fragment) (I)	phenylalanine
M103T3	103.0542	3.46	1.02	0.918	phenylalanine (fragment) (III)	phenylalanine
M116T1_3	116.0705	1.18	−1.00	0.969	proline (I)	proline
M116T2	116.0703	1.61	1.03	0.867	proline (II)	proline
M89T1	89.1071	0.98	−1.02	0.939	putrescine	putrescine
M106T1	106.0489	1.34	1.16	0.238	serine	serine
M146T1_5	146.1651	0.98	1.20	0.105	spermidine	spermidine
M112T1_4	112.1118	0.96	1.18	0.232	spermidine (fragment) (I)	spermidine
M129T1_4	129.1385	0.96	1.18	0.216	spermidine (fragment) (II)	spermidine
M365T1_4	365.1057	1.14	1.18	0.057	sugar	unidentified sugar
M144T6_3	144.0807	5.93	1.06	0.525	tryptamine (I)	tryptamine
M144T7_1	144.0806	6.71	1.07	0.542	tryptamine (II)	tryptamine

**Identifier in the discovery data set Metabolite**	**Median *m*/*z***	**Median retention time [min]**	**Directional fold change**	**Corrected p-value**	**Annotation**	**Metabolite**
M205T6_1	205.0972	5.93	1.07	0.469	tryptophan	tryptophan
M188T6_2	188.0707	5.93	1.07	0.484	tryptophan (fragment) (I)	tryptophan
M188T6_1	188.0703	6.35	1.11	0.665	tryptophan (fragment) (II)	tryptophan
M182T3_2	182.0809	2.98	1.16	0.092	tyrosine	tyrosine
M169T2	169.0353	1.68	1.36	0.001	uric acid	uric acid
M153T2_2	153.0403	1.77	1.22	0.330	xanthine (I)	xanthine
M153T2_1	153.0404	2.04	1.22	0.305	xanthine (II)	xanthine

References

1. Scherber, W. Stable Isotope Labeling to Improve Metabolite Identification in Untargeted Metabolomics of Pathogenic Bacteria. Master's thesis, Hochschule Aalen – Technik und Wirtschaft, 2020.
2. Sumner, L.W.; Amberg, A.; Barrett, D.; Beale, M.H.; Beger, R.; Daykin, C.A.; Fan, T.W.M.; Fiehn, O.; Goodacre, R.; Griffin, J.L.; Hankemeier, T.; Hardy, N.; Harnly, J.; Higashi, R.; Kopka, J.; Lane, A.N.; Lindon, J.C.; Marriott, P.; Nicholls, A.W.; Reily, M.D.; Thaden, J.J.; Viant, M.R. Proposed minimum reporting standards for chemical analysis. *Metabolomics* **2007**, *3*, 211–221. doi:10.1007/s11306-007-0082-2.
3. Tautenhahn, R.; Patti, G.J.; Rinehart, D.; Siuzdak, G. XCMS Online: A Web-Based Platform to Process Untargeted Metabolomic Data. *Analytical Chemistry* **2012**, *84*, 5035–5039. doi:10.1021/ac300698c.
